# Genetic model of the El Laco magnetite-apatite deposits by extrusion of iron-rich melt

**DOI:** 10.1038/s41467-022-33302-z

**Published:** 2022-10-17

**Authors:** Tobias Keller, Fernando Tornos, John M. Hanchar, Dorota K. Pietruszka, Arianna Soldati, Donald B. Dingwell, Jenny Suckale

**Affiliations:** 1 Stanford University, Department of Geophysics, Stanford, USA; 2 ETH Zurich, Department of Earth Sciences, Zurich, Switzerland; 3 Instituto de Geociencias (IGEO, CSIC-UCM), Madrid, Spain; 4 Memorial University of Newfoundland, Department of Earth Sciences, St. John’s, Canada; 5 North Carolina State University, Department of Marine, Earth, and Atmospheric Sciences, Raleigh, NC USA; 6 Ludwig-Maximilians University of Munich, Department of Earth and Environmental Sciences, Munich, Germany

**Keywords:** Geochemistry, Petrology, Tectonics, Volcanology, Computational science

## Abstract

Magnetite-apatite deposits are important sources of iron and other metals. A prominent example are the magnetite lavas at the El Laco volcano, Northern Chile. Their formation processes remain debated. Here, we test the genetic hypothesis that an Fe-rich melt separated from silicate magma and ascended along collapse-related fractures. We complement recent analyses with thermodynamic modelling to corroborate Fe-Si liquid immiscibility evident in melt inclusions at El Laco and present viscometry of Fe- and Si-rich melts to assess the time and length scales of immiscible liquid separation. Using a rock deformation model, we demonstrate that volcano collapse can form failure zones extending towards the edifice flanks along which the ore liquid ascends towards extrusion driven by vapour exsolution despite its high density. Our results support the proposed magmatic genesis for the El Laco deposits. Geochemical and textural similarities indicate magnetite-apatite deposits elsewhere form by similar processes.

## Abstract (web summary)

Can volcanoes erupt ore deposits? This study combines observations, experiments, and simulations to show that iron ore deposits on El Laco volcano formed by eruption of melt sourced from separation of Fe-rich melt from a silicate magma body beneath.

## Introduction

Magnetite-apatite (MtAp) deposits, also known as iron-oxide-apatite (IOA) or Kiruna-type, are a strategically important resource^1,2^. They comprise large bodies of high-grade magnetite (Fe_3_O_4_) ore enriched in Ca-Mg-(Fe)-bearing silicates (diopside, actinolite), sulphate-bearing minerals (anhydrite, scapolite), and phosphates (apatite, monazite) with, at some localities, significant concentrations of uranium, cobalt, and rare earth elements (REE). Their genesis is the subject of ongoing debate.

Based on geochemical findings, some studies have attributed the formation of MtAp deposits to hydrothermal replacement or precipitation in subvolcanic environments^3–5^. In contrast, the prevalent hypothesis of orthomagmatic to magmatic-hydrothermal ore genesis posits a formation by fracture-facilitated shallow intrusion to extrusion of an iron-enriched liquid of magmatic origin. The hypothesis is based largely on the conspicuous and well-exposed geology of the deposits at El Laco, an andesitic arc volcano in the Central Volcanic Zone, Northern Chile^6^, their unique morphology, reminiscent of basaltic lava flows and tephra deposits, as well as their positioning along interpreted collapse structures on the edifice flanks^1,6–14^. The nature and source of the ore-forming liquid, as well as the mechanics of its emplacement, however, remain contentious.

Recent evidence from melt inclusions hosted in silicate phenocrysts from El Laco andesite^15,16^, as well as from petrology experiments on similar compositions, suggest that exsolution of an immiscible Fe-rich melt from a parent andesite magma^17–19^ potentially aided by assimilation or anatexis of evaporites in the shallow crust^20^ may be the source of an iron-silicate(-phosphate-sulfate) ore liquid. In contrast, others^12,14,21^ have proposed the liquid to be a magnetite-brine suspension formed by flotation and accumulation of bubble-oxide aggregates^22^. Recent experiments confirm that bubble-oxide aggregates can form in a hot and wet andesite magma as magnetite phenocrysts heterogeneously nucleate on volatile bubbles^23^, or vice versa^24^. However, experiments also show that flotation rates of small bubble-oxide aggregates are relatively slow^24^. Bubble growth and coalescence would allow more rapid flotation, but could result in detachment of the oxide load^23^.

In this contribution, we refine a genetic model involving Fe-Si melt immiscibility^9^ and test its consistency with available evidence as well as the internal consistency of the physical processes comprised in it. We present: (i) thermodynamic modelling to demonstrate broad agreement between recent high-resolution geochemical analyses of immiscible liquids preserved in melt inclusions from El Laco^15,16^ and evidence from petrological experiments^18,19^; (ii) viscometry experiments on Fe- and Si-rich immiscible melts to constrain time- and length-scales of immiscible liquid separation in a sub-volcanic magma body; (iii) a volcano deformation model to show that collapse fractures emerging around a deflating magma body can provide extraction pathways for extrusive ore emplacement; and (iv) a scaling analysis of bubbly liquid flow confined to a fracture to argue that, once pressed into fractures during collapse-related slip events, volatile exsolution and bubble expansion can drive an Fe-rich melt to eruption despite its high density.

Our results show that the proposed genetic model can explain observational and experimental evidence by a sequence of internally consistent petrological and mechanical processes. The geochemical and geological similarities between El Laco and MtAp deposits elsewhere suggest that ore formation by intrusive to extrusive emplacement of an Fe-rich melt sourced from liquid immiscibility may apply to the genesis of Kiruna-type deposits more generally. Our genetic model has important ramifications for understanding other orthomagmatic mineral systems, for resource exploration, for magma differentiation at continental arcs, and for comparative planetary research.

## Results and discussion

### Geology of the El Laco deposits

**Fig. 1:**
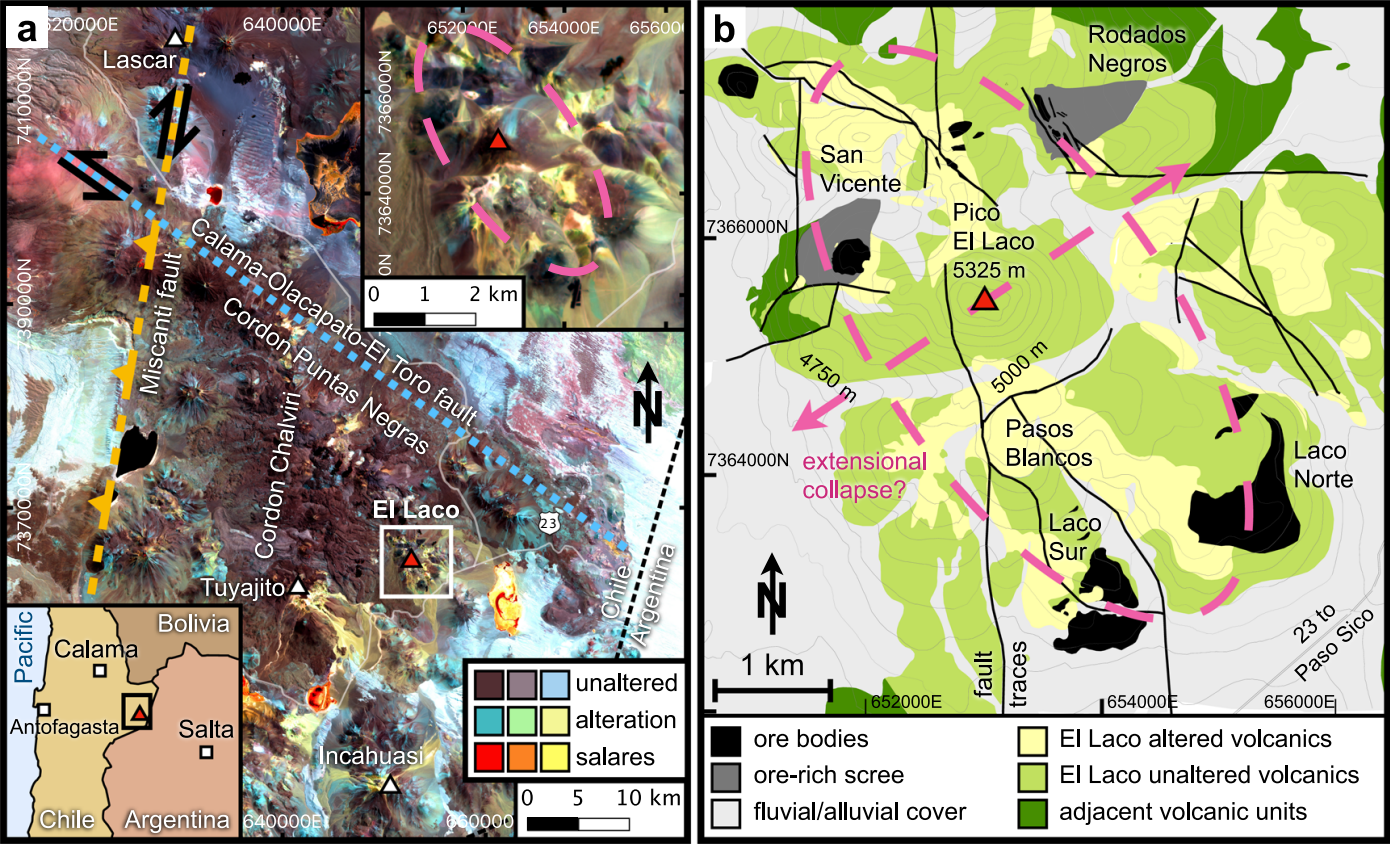
Regional tectonic and local geologic context of El Laco volcanic centre, Northern Chile. **a** Sentinel-2 infrared pseudo-colour image (RGB: bands 8, 11, 12) highlights areas of unaltered volcanics and alluvial cover (brown-purple-lavender), altered volcanics (teal-light green-beige), and salares (red-orange-yellow). Other volcanoes in the vicinity are labelled by white triangles. NS-trending right-lateral transpressive Miscanti fault (amber dashed) and NW-SE-trending left-lateral Calama-Olacapato-El Toro fault (light blue dotted) dominate regional tectonics; inset panels show geographic context (lower left) and magnified view of El Laco complex (top right). **b** Simplified geological map of the El Laco volcanic complex. Ore bodies are situated along interpreted collapse structure (pink dashed) aligned between NS and NW-SE trending regional lineaments and interpreted NE-SW least compressive stress direction (pink dashed arrows). Sentinel-2 (ESA) image courtesy of the U.S. Geological Survey https://earthexplorer.usgs.gov/. Figure 1b is based on a map in ref. 9. 10.5382/econgeo.2017.4523.

El Laco is a relatively well-preserved, 5.3–1.6 Myr old^25^ stratovolcano situated in the Central Volcanic Zone of Northern Chile. Figure 1a shows that El Laco is located ~8 km south of the NW-SE-trending Cordon Puntas Negras, and the same distance east of the N-S-trending Cordon Chalviri volcanic chains. The regional tectonic is dominated by the NW-SE-trending left-lateral Calama-Olacapato-El Toro fault^26^ and the N-S-trending right-lateral transpressive Miscanti fault^27^ (Fig. 1a). The regional salares (salt lakes, red to yellow in Fig. 1a) have been interpreted as indicative of basin-and-range type crustal deformation^28^ with shortening in NW-SE and extension in NE-SW direction (Fig. 1b).

**Fig. 2:**
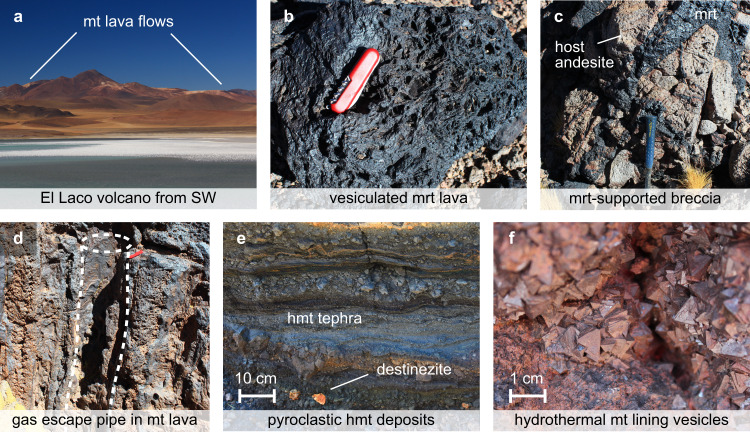
Examples of field evidence for extrusive ore formation. **a** El Laco volcano with magnetite (mt) lava flows seen from SW; **b** vesiculated martite (mrt, haematite pseudomorph after mt) lava; **c** volcanic breccia with veins supporting fragments of altered andesite; **d** meter-scale gas escape pipe in mt lava flow; **e** mt tephra layers of ash and lapilli with inclusions of destinezite (hydrated Fe-phosphate-sulfate); **f** late-stage hydrothermal mt phenocrysts lining walls of larger vesicles.

The El Laco deposits comprise six massive, strata-bound magnetite bodies interbedded with andesitic lava flows, with only a few stratigraphically younger andesite units overlaying the deposits^9,29^. The extent of erosion remains uncertain, but at least some of the deposits appear to have remained subaerial since their emplacement, pointing to a late emplacement age within the life span of the volcanic centre^9^. The ore bodies are situated along what has been interpreted as a collapse structure tracing a NW-trending ellipse around the central edifice^7,9,14^ (Figs. 1b, 2a). The morphology and textures of the ore are reminiscent of effusive to explosive volcanic emplacement of basalt^1,6–11,13,29,30^ (Fig. 2b–e). Some units show lava flow-like textures with pronounced vesiculation and cm- to m-scale gas escape pipes^11,29,30^. Others present strata of magnetite-rich tephra including bombs^10^, scoria, and ash^11,13^. At Laco Sur, Laco Norte, and Rodados Negros (Fig. 1b), subvertical feeder systems are exposed, comprising magnetite-filled dykes and veins, magnetite-supported breccias, and small maar-diatreme structures cutting through andesite lava^9^. The ore bodies are surrounded by discrete zones of alkali-calcic alteration with K-feldspar, diopside, magnetite, and scapolite, as well as large areas of andesite pervasively affected by steam-heated acidic alteration^9,25^. There is evidence from drill cores of a stock-work of magnetite-rich and apatite-bearing dykes with veins of magnetite-clinopyroxene-anhydrite-scapolite assemblages beneath the steam-heated acidic alteration zone at the Pasos Blancos locality at El Laco^9,25^.

In summary, the geology of the MtAp deposits at El Laco is consistent with ascent along collapse fractures and effusive to explosive emplacement of an over-pressured, gas-rich liquid accompanied by hydrofracturing, copious magmatic outgassing and related hydrothermal wall rock alteration.

### Ore melt from Fe-Si liquid immiscibility

**Fig. 3:**
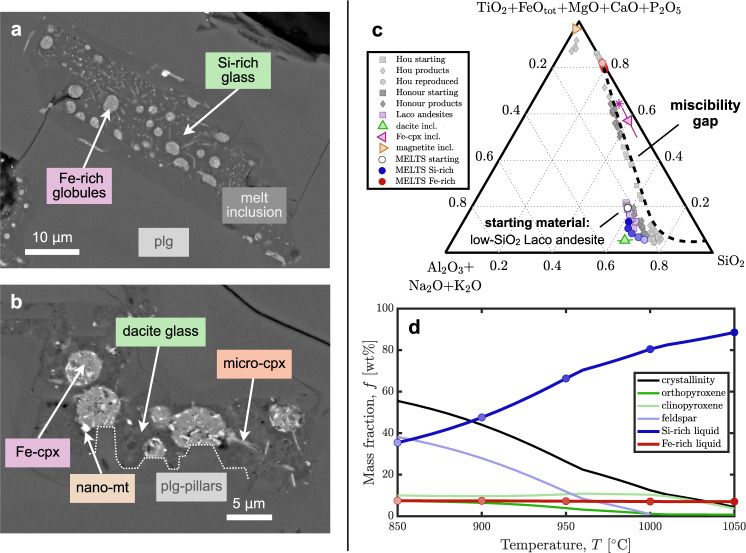
Liquid immiscibility in El Laco andesite. Back-scattered electron images of plagioclase-hosted (plg) melt inclusions from ore-hosting andesites; **a** Fe-rich globules in Si-rich matrix; **b** higher magnification image shows high-SiO_2_ dacite glass matrix with some clinopyroxene micro-phenocrysts (micro-cpx), and Fe-rich globules comprised of Fe-clinopyroxene (Fe-cpx) with some nanocrystalline magnetite (nano-mt); plagioclase shows pillar structures indicative of melt unmixing during crystallisation^19^. Modelling of closed-system isobaric cooling in alphaMELTS shown at 50 °C intervals from 1050 °C (darker) to 850 °C (lighter) in (**c**) predict Si-rich melt (blue) similar to analyses of high-SiO_2_ dacite glass (green, error bars ± 1 standard deviation), Fe-rich melt (red)^16^ on mixing line between analyses of Fe-cpx (pink, error bars ± 1 standard deviation) and magnetite (amber) from inclusions^15^, with reconstructed Fe-cpx-mt globules from 80% Fe-cpx + 20% mt shown as asterisk; approximate miscibility gap (dashed) according to experimental results^18^ (grey symbols). Modelled phase fractions (**d**) show similar crystal types and abundances to observations and experiments.

Evidence for the nature and source of the ore liquid derives from melt inclusions^15,16^ hosted in plagioclase, clinopyroxene, and orthopyroxene phenocrysts recovered from andesite lava flows at El Laco. The melt inclusions shown in Fig. 3a contain rounded globules of an Fe-rich composition embedded within a Si-rich matrix. Upon closer inspection of the globules (Fig. 3b), nanocrystalline magnetite is found embedded in a matrix of Fe-clinopyroxene composition. By modal analysis (see Supplementary Figs. 1 and 2) of these inclusions, we find proportions of ~18–24% Fe-rich to ~76–82% Si-rich material, and of ~20% magnetite to ~80% Fe-clinopyroxene within globules, consistent with the more extensive study by ref. 16. Based on their morphology and contrasting compositions, these inclusions have been interpreted as evidence of the spontaneous unmixing of an andesitic parent magma into Fe-rich and Si-rich melts^9,15,16^. Experiments on a similar range of compositions^18,19^ support that interpretation.

The compositions of the immiscible liquids have previously been analysed by electron-probe micro-analysis (EPMA)^15^. However, the small size of Fe-rich globules and regions of Si-rich matrix free from phenocrysts suggests that the measurements were likely biased towards the surrounding Si-rich glass in the former or included more than one phase in the latter.^16^ re-analysed the same thin section using a field emission gun electron-probe micro-analyzer (FEG-EPMA) to spatially resolve these materials and obtain precise and accurate compositional data. The estimated horizontal spatial resolution of their analyses is ~900 nm compared to >2000 nm in ref. 15. In Table 1, we list average compositions and range of 24 FEG-EPMA measurements of the Fe-clinopyroxene composition within the globules (not including nano-magnetite), and 21 of the high-SiO_2_ dacite glass in the matrix (not including clinopyroxene micro-phenocrysts) from ref. 16.

**Table 1:** Analysed compositions of immiscible liquids in melt inclusions and model compositions for parent andesite at El Laco

Oxide	High-SiO_2_ dacite^a^	Fe-cpx^a^	Fe-rich melt^b^	Low-SiO_2_ andesite^c^
SiO_2_	67.94 (1.40)	40.48 (2.46)	32.47	57.50
TiO_2_	^*^0.25 (0.02)	5.59 (2.18)	4.97	0.86
Al_2_O_3_	18.05 (0.76)	1.89 (0.53)	2.01	16.31
FeO_tot_	4.32 (0.22)	24.35 (4.39)	36.16	6.73
MgO	^*^0.06 (0.01)	13.48 (2.33)	11.16	4.43
CaO	0.80 (0.60)	12.10 (4.51)	9.71	6.78
Na_2_O	4.80 (1.26)	0.68 (0.35)	0.55	3.45
K_2_O	8.99 (2.63)	0.34 (0.11)	0.27	1.95
P_2_O_5_	^*^0.20 (0.02)	2.26 (1.29)	1.81	0.22
H_2_O	–	–	–	1.54
Sum	104.90	101.17	100.00	99.78

^a^Analyses of melt inclusions in El Laco andesite from ref. 16; shown are mean (standard deviation), values marked ^*^ are at detection limit.

^b^Reconstructed composition from 80% of mean Fe-cpx from ref. 16 + 20% mean Type 2a magnetite from ref. 15 normalised to 100%.

^c^Average of five lowest SiO_2_ host andesite samples analysed by ref. 9.

Figure 3c shows the averaged major oxide compositions of the Fe-clinopyroxene in the globules and the SiO_2_-rich dacite glass of the matrix, together with the average composition of magnetite from melt inclusions (Type 2b from ref. 15) and a range of whole-rock compositions of the host andesite^9^. On the ternary space of SiO_2_ vs. Al_2_2O_3 _+ Na_2_O + K_2_O, vs. FeO_tot _+ MgO + TiO_2 _+ CaO + P_2_O_5_, the dacite glass plots near 65 wt% SiO_2_, 5 wt% Fe-rich, and 30 wt% Al-alkali components, whereas the Fe-clinopyroxene composition plots near 40 wt% SiO_2_, 55 wt% Fe-rich, and 5 wt% Al-alkali components. The re-analysed Fe-clinopyroxene composition is significantly more Fe-rich and Si-poor than previously reported^15^. Adding 20% magnetite to 80% Fe-clinopyroxene we reconstruct an Fe-melt composition within the analysed melt inclusions with 32.5 wt% SiO_2_ and 36.2 wt% FeO_tot_ (see Fig. 3c, and Table 1).

Petrography of parent andesite^9,15^ and experimental petrology on similar starting compositions^18,19^ suggest that liquid immiscibility occurrs during crystallisation on an interval of ~10–50% crystallinity comprised predominantly of plagioclase, with some clino- and orthopyroxene, and accessory magnetite. Based on FEG-EPMA analyses^16^ and previous analyses of plagioclase, clinopyroxene, and orthopyroxene in the parent andesite^15^, as well as magnetite in melt inclusions^15^, we find by least-squares fit that an average El Laco andesite composition (see Table 1,^9^) can be reconstructed from 5.13 wt% Fe-rich melt + 39.7 wt% high-SiO_2_ dacite + 31.9 wt% plagioclase + 11.1 wt% orthopyroxene + 12.2 wt% clinopyroxene. This corresponds to proportions of 55.2–44.8 wt% phenocrysts to total melt, and 11–89 wt% Fe-rich to Si-rich melt (see Table 1). Based on the reconstruction constrained by melt inclusion analyses, ~1940 Mt of this melt derived from ~38 Gt of parent andesite would suffice to generate the 700 Mt magnetite deposits at El Laco. That amount of parent magma corresponds to ~15 km^3^, or the volume of a spherical body of 1.5 km radius.

The recent experiments by ref. 19 on compositions close to El Laco andesites and by ref. 18 on slightly more mafic and Fe-rich compositions both found robust evidence of Fe-Si liquid immiscibility. They describe an Fe-silicate to Fe-Ca-P-rich melt exsolving from a dacitic to rhyolitic Si-rich residual magma (grey symbols, Fig. 3c).^19^ find that the exsolution occurs during plagioclase crystallisation below ~1000 °C, aided by the formation of Fe-rich compositional boundary layers around plagioclase crystals and evidenced by what they term ‘pillars’: angular protrusions of plagioclase growth in the vicinity to growing droplets of exsolved Fe-rich melt. The same structures are observed in El Laco melt inclusions (Fig. 3b). While both studies document a widening of the miscibility gap with progressive cooling, ref. 18 demonstrate that an increase in H_2_O, P_2_O_5_, and/or ${f}_{{{{{{{{{\rm{O}}}}}}}}}_{2}}$ can produce Fe-P- to Fe-Ca-P-rich melts of up to 40% FeO_tot_ and <10% SiO_2_ + Al_2_O_3_ + alkali oxides. Similar immiscible P-rich melts have also been documented in experiments by refs. 31, 13, and 32.

We use the alphaMELTS thermodynamic software (v. 1.9)^33–35^ with the option to detect immiscible liquids^36^ to model immiscible melt compositions for El Laco and compare to melt inclusion analyses and experimental results. The alphaMELTS liquid immiscibility model has not been specifically calibrated for this compositional space. However, the reasonable fit to observations demonstrates the utility of such models, particularly once specifically calibrated for this task. We compose starting compositions from averages of El Laco andesite analyses in ref. 9 (Table 1), and perform calculations of closed-system, isobaric equilibration, from 1050 to 850 °C, at 125 MPa (ca. 5 km depth), at oxygen fugacities ~1.5–3 log-units above quartz-fayalite-magnetite (QFM), and with 1–5 wt% H_2_O added. The model results for mean low-SiO_2_ El Laco andesite + 3 wt% H_2_O (Table 1; Fig. 3c, d) show an Fe-rich melt in composition between Fe-clinopyroxene and magnetite coexisting with a dacitic to rhyolitic Si-rich melt and up to 35% phenocrysts of plagioclase, clino-, and orthopyroxene. The melts evolve to more Fe- and Si-rich compositions along the cooling path from the andesite liquidus at ca. 1050 °C down to 850 °C, at the lower bound of crystallisation temperatures estimated for the inclusion-hosting mineral assemblage^29^. The alphaMELTS model predicts the stability of ~7 wt% Fe-rich melt at ~75 wt% FeO_tot_, ~20 wt% SiO_2_, and ≪1 wt% of aluminium and alkali oxides.

The alphaMELTS model predicts the Si-rich melt composition reasonably well compared to both analyses^16^ and experiments^18,19^ (see Supplementary Figs. 3–5). However, it over-predicts the Fe- and under-predicts the Si-, Mg-, Ca-, P-, and water contents of the Fe-rich melt. The modelled miscibility gap appears wider than in melt inclusion analyses and experiments. The Si-rich melt tends towards a dacitic to rhyolitic composition with cooling, while the Fe-rich melt falls on an apparent mixing line between Fe-clinopyroxene and magnetite. Our reproduction of the results of ref. 18 shows systematic offsets, resulting in less than half the mass but more than double the Fe-content in the Fe-rich melt compared to experiments. Nevertheless, the model demonstrates broad internal consistency between experiments and observations. If appropriately recalibrated, the model has the potential to become a valuable tool for the study of orthomagmatic ore liquids sourced from liquid immiscibility.

### Ore melt separation from parent magma

Based on geochemical analyses, experimental petrology, and our thermodynamic modelling we hypothesise that magma unmixing at El Laco occurred during cooling and crystallisation in a subvolcanic magma body. To be emplaced as an ore body, however, the Fe-rich liquid must first separate from its parent magma. The high density (3500-4000 kg/m^3^) suggests that the Fe-rich melt may segregate from the Si-rich magma (~2400 kg/m^3^) and collect along the base of the reservoir.

**Fig. 4:**
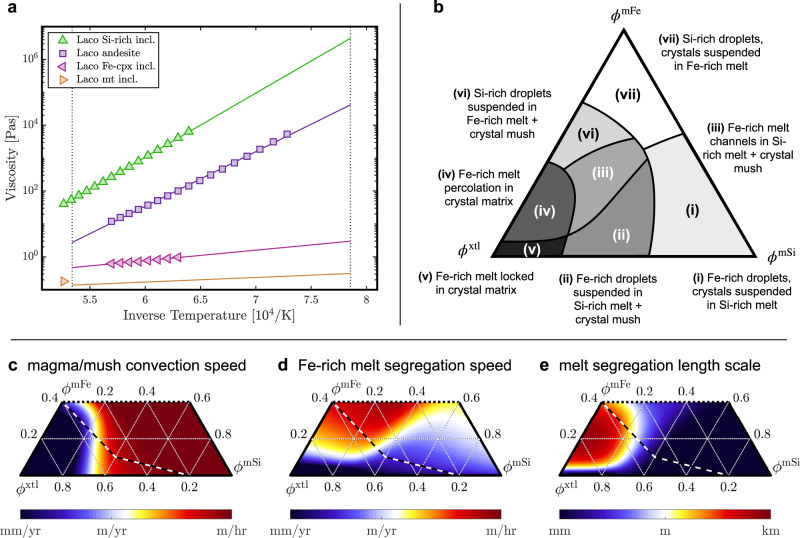
Viscometry and scaling analysis pertaining to ore melt separation from parent magma body. **a** Viscometry results (symbols) with fitted model (lines) for rhyolite (green), andesite (purple), Fe-clinopyroxene (pink), and magnetite (amber) compositions (see Table 1 and Supplementary Table 1); **b** regime diagram of three-phase flow between Si-rich melt (*ϕ*^mSi^), Fe-rich melt (*ϕ*^mFe^), and phenocryst (*ϕ*^xtl^) phases transitioning from droplet settling at high melt content to melt percolation at intermediate to high crystallinity. **c**–**e** Results of the scaling analysis giving the characteristic speed of phase buoyancy-driven magma/mush convection (**c**), the speed of Fe-rich melt segregation from the silicate magma/mush (**d**), and the characteristic length scale of Fe-rich melt segregation (**e**).

To quantify the time- and length scales of Fe-rich melt separation, we perform viscometry experiments on a range of relevant melt compositions, the results of which are shown in Fig. 4a. We use a log-linear model fit to the measured data to extrapolate the viscosity measurements to the relevant temperature range of exsolution (~1000–900 °C) and find that the parent andesite melt may have an approximate viscosity of ~1–5 × 10^4^ Pas, the Si-rich end-member melt of ~1–5 × 10^6^ Pas, and the Fe-rich end-member melt of ~0.5–2 Pas, depending on the degree of Fe-enrichment bounded between the magnetite and Fe-clinopyroxene compositions observed in melt inclusions. The measurements are upper bounds on viscosity since experiments had to be conducted without the addition of volatiles due to present technology limitations; the natural system, however, shows signs of copious outgassing and hence likely has been volatile-rich at depth.

In experiments^19,37^ and melt inclusions^15,16^, the Fe-rich melt presents as sub-millimetre-sized droplets with high wetting angles to the dominant phenocryst phases of plagioclase and clinopyroxene. Modelling of droplet settling in magmatic emulsions suggests that droplet collision and coalescence provide a way for initially very small droplets to grow and segregate rapidly relative to the time scale of magma body cooling^38^. Droplet settling may be further enhanced by convective flow driven by lateral heterogeneities in droplet and crystal concentrations^39^. As crystallinity increases, droplet settling will initially be hindered by crystals increasing the effective viscosity of the magma. Conceptual^40^, analogue^41^, as well as numerical modelling^42^ of low-viscosity, high-wetting angle fluids segregating from crystallising magma suggest that at intermediate crystallinity (~40–60%), fluid segregation will transition from droplet settling to percolation along a drainage network of interconnected channels potentially enhanced by capillary fracturing. The mechanics of this problem are analogous to that of a magmatic volatile fluid escaping from magma during shallow degassing^41,42^, although in our case the segregating fluid is negatively buoyant and somewhat higher in viscosity.

We use the multi-phase model framework of ref. 43 to perform a scaling analysis of the characteristic rates and length scales of Fe-rich melt segregation from the crystallising parent magma. The analysis relies on a tentative calibration of phase connectivity as a function of volumetric phase fraction encapsulating the hypothesised transition from droplet settling to channelised percolation^40–42^. Figure 4b shows the three-phase regime diagram arising from the chosen calibration (see also Supplementary Fig. 6). The scaling analysis assumes melt viscosities of 10^5^ and 1 Pas and densities of 2400 and 4000 kg/m^3^ for the Si- and Fe-rich melts, and a characteristic size of local-scale phase constituents of 1 mm. The results in Fig. 4c show that droplet settling carried by convective flow may occur at rates up to ~m/h at crystallinities <50%. If the fraction of Fe-rich melt increases to ~20% by continued exsolution and accumulation, the speed of Fe-melt segregation by channelised percolation will increase to ~m/h (Fig. 4d), establishing a drainage network on a natural segregation length scale increasing from order <1 to >100 m (Fig. 4e). Hence, in a parent magma body of ~500  m thickness, the time scale of initial separation by convection-enhanced droplet settling is ~500 yr, with the time scale of ensuing channelised percolation of similar duration. In comparison, the characteristic time of conductive cooling for the same magma body is ~8 kyr. Channelised percolation may well be further enhanced by capillary fracturing^41^ or decompaction weakening^44^. This scaling analysis therefore suggests that ore melt separation from the parent magma body is mechanically viable during the cooling of a subvolcanic magma body at El Laco.

### Volcano collapse fractures

For an Fe-rich melt pooled at the base of a subvolcanic magma body to rise to the surface suitable extraction pathways and a driving force must be available. Addressing the former condition first, it has been suggested that the melt appears to have extruded along volcano collapse fractures^14,24^. The location of the ore bodies on the edifice along with locally mapped fault traces (Fig. 1b) indicate a SE–NW trending elliptical collapse structure^7,9^, while regional tectonic lineaments, and proximity of several salares (Fig. 1a) suggest its semi-major axis is aligned at right angles to a NE-SW trending least compressive stress direction.

We use a custom-built model of long-term visco-elastic/brittle-plastic volcano deformation^45,46^ to evaluate the conditions under which collapse fractures extend from the base of a subvolcanic magma body to its flanks. The model domain represents an idealized 2-D section across the volcanic edifice, a two-layer subsurface geology of young volcanic units above a slightly less rigid metasedimentary basement, and the subvolcanic magma body imposed as a weak inclusion beneath the edifice. We test a range of magma body deflation and inflation rates, and extensional and compressive regional tectonic stress conditions (see Supplementary Fig. 7).

**Fig. 5:**
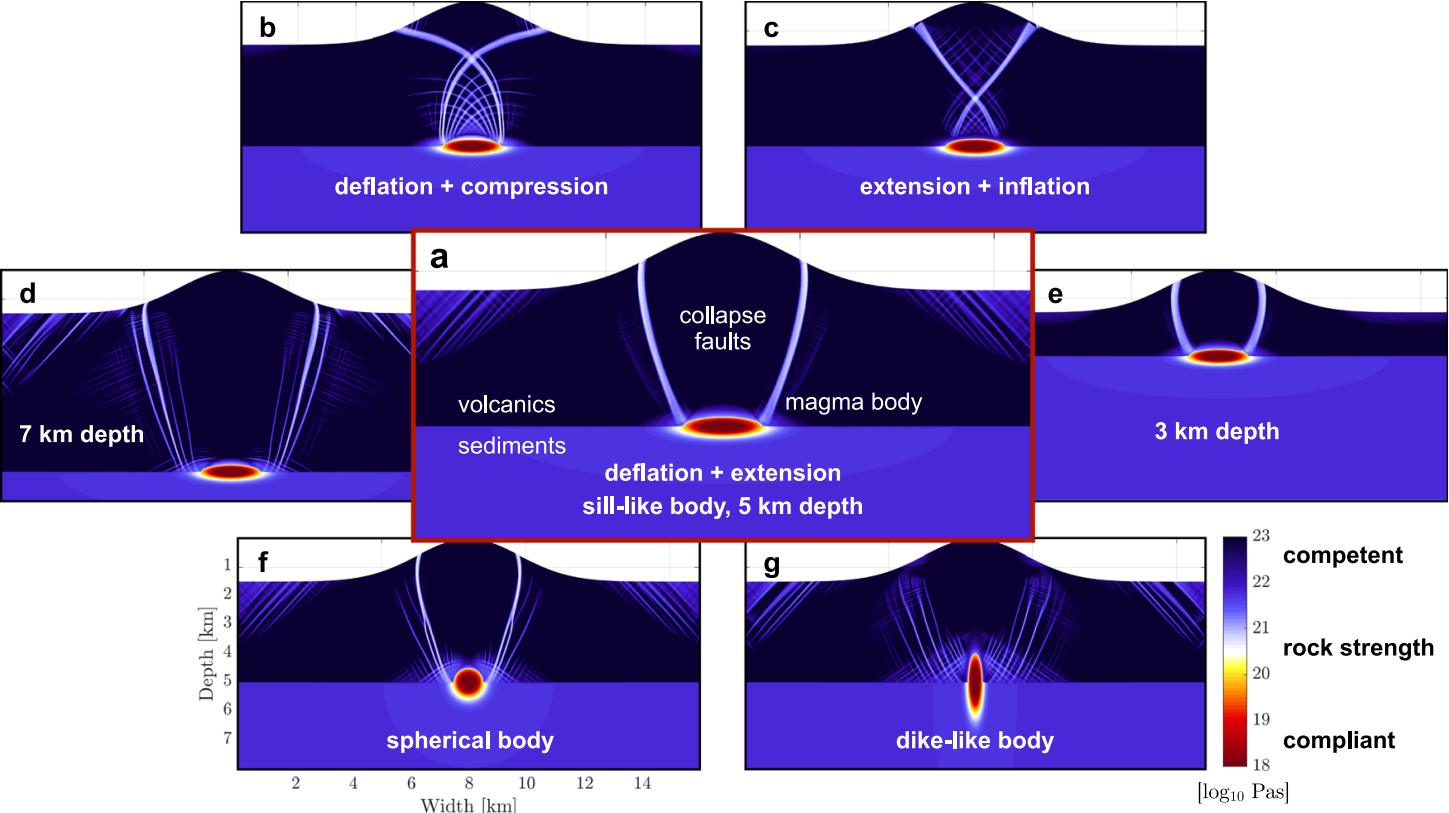
Results of volcano deformation models show emerging collapse fracture geometries. **a** Best-fit case of deflating magma body under tectonic extension, steep failure zones extend laterally from base of magma body to edifice flanks; **b** for deflation combined with compression, and **c** for extension combined with inflation, steep cross-cutting fracture zones emerge from roof of magma body. Favourable failure geometry is robust across magma body depths of 7 km (**d**), 5 km (**a**), and 3 km (**e**), and for a spherical body (**f**), and oblate sill (**a**), but not for a prolate dyke (**g**). Colour map shows effective visco-plastic rock strength, with warm colours indicating compliant and deforming, cold colours, competent and rigid rock.

Figure 5 shows a selection of model results from this study, with the best-fit scenario highlighted in the centre (Fig. 5a). For a sill-like magma body at a depth of 5 km, a combination of mild deflation at a rate of ~10^−13^ s^−1^ characteristic of magma cooling and crystallization, the topographic load of a 1500 m edifice, and moderate tectonic extension rate of ~10^−15^ s^−1^ produces the best-fit outcome: two steep failure bands extending from the base and lateral extremes of the body towards the lower edifice flanks.

Our results are robust across a range of geometries, rock properties, and imposed deformation rates. Similar failure zones emerge for magma body depths of 3–7 km (Fig. 5a, d, e) and for spherical and oblate bodies, but not for dyke-shaped ones (Fig. 5a, f, g). Regional extension in the absence of magma deflation causes shallower failure zones extending from the roof rather than the base of the reservoir (see Supplementary Fig. 7). An inflating magma body or regional compressive tectonics produces failure zones connecting to the roof of the magma body and crossing over along their path to the surface (Fig. 5b, c). Mild deflation due to thermal contraction is likely not sufficient to cause failure in the absence of tectonic stress, but increased deflation rates, e.g., due to magma extraction or rapid outgassing, would be (see Supplementary Fig. 7). The model therefore suggests that rock failure induced by moderate magma body deflation and tectonic extension can provide fractured ascent pathways for an Fe-rich melt in agreement with surface geology and regional tectonics.

Whereas this model simulates collapse fractures as long-term failure zones, volcano collapse in natural systems likely occurs as intermittent seismic events with finite slip along fractures. At a deflation rate consistent with our model, the magma reservoir would deflate at a rate sufficient to accumulate elastic strain for a collapse event of 10 cm slip distance within ~10 yr. During such hypothesised collapse-related slip events along fractures of a geometry as in our best-fit model (Fig. 5a), the edifice will press down onto the roof of the magma reservoir and hence increase the magma pressure within. Due to their large viscosity contrast (up to 6 orders of magnitude), the Fe-rich melt will respond to the collapse-induced pressure jump at orders of magnitude faster rates than the Si-rich residual magma. To relieve the pressure jump, the melt can be expected to exploit available fracture pathways and escape the pressurised reservoir. According to the deformation model, the ore melt will likely invade fractures sloping upwards from the lateral base of the magma body.

### Vapour-driven ascent to extrusion

We use scaling analysis to assess the effects of a collapse-related slip event on the magma reservoir and the consequent expulsion and ascent of the ore melt (all symbols, values, and formulas listed in Supplementary Table 3). Assuming an idealised collapse structure of truncated elliptical cone shape with a minor axis as in our best-fit deformation model and a major axis twice that length, a slip distance of *d*_*s*_ = 1–10 cm along the entire fracture surface releases a seismic moment magnitude of *M*_*w*_ = 4.5–5.9. This is likely an upper estimate since slip might only occur on a portion of an existing collapse structure at one time. Lowering the roof of a 400–600 m thick sill-like reservoir by *d*_*s*_ will cause a volumetric strain of *υ*_0_ = 1.7 × 10^−5 ^− 2.5 × 10^−4^ and generate a pressure jump of Δ*P* = *K*_0_*υ*_0_ = 0.02 − 2.5 MPa, depending on the effective bulk modulus of the magma reservoir, *K*_0_ = 1 − 10 GPa, (the alphaMELTS model above gives ~5 GPa). We approximate liquid invasion of collapse-related fractures by plane-Poiseuille flow along a smooth and rigid fracture plane of constant, narrow opening. We estimate that the collapse-related pressure jump drives a liquid of a viscosity *μ*_0_ = 0.01 − 1 Pas into a fracture of opening *H*_0_ = 1 − 10 mm at speeds of *w*_in_ = 0.1 μm/s − 7m/s. At intermediate values on the estimated range of injection rates melt ascent to the surface would take tens of days.

The hypothesised Fe-rich melt, however, is unlikely to continue ascending at the rate of initial injection. Once it is driven into a steepening fracture zone at depth, it will experience decompression by flowing away from the imposed pressure jump, as well as by ascending against gravity. Assuming water-saturated conditions typical for shallow arc magma reservoirs, experiments^18^ complemented by our thermodynamic modelling suggest a range of 0.1–2 wt% H_2_O (and potentially other volatiles) dissolved in the Fe-rich melt at reservoir depth. Assuming the volatile solubility decreases upon decompression as in silicate magmas (first boiling)^47^, we expect a magmatic volatile phase to exsolve into bubbles as the Fe-rich melt ascends. Crystallization of magnetite upon cooling may further facilitate volatile saturation and exsolution (second boiling). Exsolved volatile bubbles will further expand under decompression, particularly if the volatile phase is a compressible vapour. Based on oxygen stable isotope analyses of the El Laco MtAp deposits and associated alteration zones, ref. 8 infer that volatile exsolution during eruptive emplacement of the ore melt at El Laco likely produced both an intermediate density Cl-rich brine, and a low density vapour rich in S, F, and P. With decreasing pressure, the volume fraction of the gaseous vapour should increase substantially (>99 vol%), with only a small fraction of a hyper-saline brine to hydro-saline melt remaining. While the former has been used to explain the abundant steam-heated acidic alteration, the latter has been interpreted as responsible for the pervasive high-temperature (>700 ^∘^C) alkali-calcic alteration of the andesites hosting the deposits^9^.

The volume increase from volatile exsolution and bubble expansion confined within a thin, rigid fracture can be released by moving the liquid further up along the fracture at increasing speed. Fracture-bound flow of a bubbly liquid is a physical process of considerable complexity^48^. Here we limit our considerations to scaling arguments drawn from an idealised model of the pressure-driven flow of a bubbly liquid along a pre-existing, thin, rigid fracture based on the Mixture Theory^49^ framework for multi-phase flows of ref. 43. The model has the ascent speed of the bubbly liquid grow along the fracture as a function of volume increase by exsolution of new vapour bubbles and expansion of existing ones. The equation for the ascent speed (see Methods) admits exponential solutions where the initial ascent speed at the fracture inlet, itself driven by collapse-related overpressure in the magma reservoir, grows significantly over an e-fold growth length, *λ* (distance over which solution grows by factor *e* = 2.79). Dividing the fracture length, *L*_0_, by that length scale we form a dimensionless group of parameters,

1 $${{\Lambda }}=\frac{{L}_{0}}{\lambda }={L}_{0}\left[{\phi }_{0}(1-{\phi }_{0}){\rho }_{0}^{\ell }{g}_{0}\cos ({\alpha }_{0})\left({\beta }_{0}+\frac{{\gamma }_{0}}{{\rho }_{0}^{g}}\right)\right]\,,$$

which expresses the potential for exponential growth of ascent speed as a function of pertinent model parameters. If the growth length is smaller than the fracture length (*λ*_0_ < *L*_0_; Λ > 1) the model predicts that volume expansion drives the ore liquid towards the surface despite its high density. The growth number is a function of the volatile phase compressibility, *β*_0_, the volatile solubility gradient in the ore liquid, *γ*_0_, the density contrast between the ore liquid and volatile vapour phases, ${\rho }_{0}^{\ell }/{\rho }_{0}^{g}$, and gravity acting on the fracture inclined at angle *α*_0_ to the vertical, ${g}_{0}\cos ({\alpha }_{0})$.

For an appropriate range of parameter values the growth number assumes values of order 1–100. The upper end of parameter estimates is justified if the volatile phase is a vapour of close to ideal gas behaviour, and if volatile solubility is of order 1 wt% at reservoir depth. Considering the force balance of pressure gradients driven by volume expansion and viscous friction of the liquid flowing between static fracture walls suggest that volume expansion and growing ascent speed will entail increasing liquid over-pressure along the fracture. A scenario of significant liquid overpressure during emplacement is consistent with the massive magnetite found within dykes, breccias, and maar-diatreme structures observed at El Laco. The feedback between fracture-bound extrusion, pressure generation by volume expansion, and pressure release by hydrofracturing, may explain the localisation of ore liquid extrusion to a few discrete vent locations around the inferred elliptical collapse structure. Moreover, rapid heat loss to the fracture walls would lead to flow contraction from planar to cylindrical conduit geometries.

This model assumes flow of a liquid bearing a constant fraction of small, uniformly distributed bubbles along a pre-existing, rigid, liquid-filled fracture. These simplifying assumptions may be consequential, particularly because we exclude a number of processes that may dampen the growth of ascent speed and liquid over-pressure along the fracture: In very narrow fractures, capillary forces may resist liquid invasion due to high wetting angles between the ore liquid and host rock minerals^19^; the escape of the volatile phase by bubble segregation from the carrier liquid or vapour escape by porous flow through fracture walls may dampen volume expansion; turbulence may increase resistance to flow as speed increases; elastic and/or plastic compliance of the fracture walls and ongoing hydrofracturing may dissipate the liquid over-pressure driving flow; finally, liquid extraction in a natural system will not follow one straight and smooth fracture but rather pass through an arduous, branching fracture network with generally higher resistance to flow. Furthermore, the bubble fraction will not remain constant as in our simplified model; instead, bubbles nucleate, grow by exsolution, expand under decompression, and collide and coalesce with nearby bubbles. As a result, bubble fraction will generally grow along the fracture, which may further amplify the ascent speed. Bubble size will not remain constant either in space or time; growing bubbles may rise more rapidly and escape the liquid or grow to the size of the fracture opening and thus hinder flow. As a result, the flow behaviour of the bubbly liquid will be complex and likely becomes intermittent or wave-like.

Taking these limiting factors into account, ascending ore melt is unlikely to always breach the surface. It can be expected that, at times, melt stalls and solidifies as dykes or sills in the subsurface, such as the dykes drilled into beneath Pasos Blancos at El Laco^9,25^. We note that the bulk assemblage in some magnetite-diopside-anhydrite pegmatite veins is closer to the Fe-silicate melt compositions we report from exsolved droplets in andesite melt inclusions than to the highly Fe-enriched composition of the massive magnetite dykes and strata-bound ore deposits at El Laco. Overall, the evidence at El Laco suggests that magma unmixing may have produced a range of variably Fe-enriched melts emplaced on a spectrum from intrusive to extrusive features.

### Genetic model synthesis

**Fig. 6:**
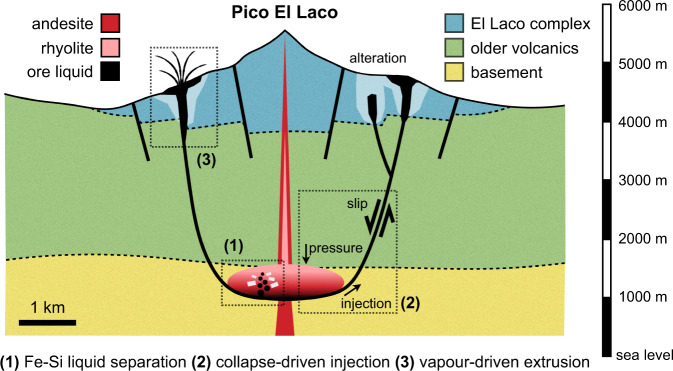
Genetic model of magnetite-apatite deposits at El Laco. (1) exsolution and gravitational separation of immiscible Fe-P-rich melt, (2) driven into collapse fractures during slip events, and (3) extruded by volume-generating feedback of vapour exsolution and bubble expansion upon ascent.

Based on geological and geochemical observations interpreted according to our thermodynamic calculations, viscometry and scaling analysis of ore melt separation, volcano deformation modelling, and scaling analysis of bubbly fracture flow, we synthesise a process-based, internally consistent genetic model for the MtAp deposits at El Laco. Figure 6 shows a schematic representation of our model synthesis.

An important open question remains: what best explains the apparent discrepancy between the highly magnetite-enriched composition of arrested dykes and strata-bound ore bodies, the magnetite-diopside-anhydrite composition of pegmatite veins, and the Fe-clinopyroxene-magnetite globules interpreted as immiscible droplets in melt inclusions? Perhaps the andesite lavas with unmixing only evident in melt inclusions represent the system before conditions were met for massive magnetite mineralisation? Or perhaps liquid immiscibility may have been more pronounced in some magma batches than others? One factor that may explain the latter are various degrees of crustal assimilation, potentially linked to various magma residence times in the shallow crust. The basement beneath El Laco comprises early Paleozoic metasediments including ironstone^50^, and phosphorite^51^. Both have the potential to enhance immiscibility by increasing Fe and P abundances in the system^18,37^. The strontium isotope composition of El Laco units^9^ suggests that the magma may have assimilated crustal lithologies during transport and storage at ~4–6 km depth beneath the edifice; the signature of contamination is found to be strongest in the ore deposits themselves compared to host andesites. The recent discovery of sulphate-rich Fe-silicate melts in melt inclusions from the arrested dykes at Pasos Blancos^20^ supports the hypothesis that assimilation or anatexis of evaporite layers in the shallow crust was instrumental in promoting ore genesis at El Laco.

A second open question is the fate of the Si-rich conjugate to the ore melt after unmixing and separation. Liquid immiscibility should have produced considerable volumes of dacitic to rhyolitic magma. Ref. 30 report a relatively young rhyo-dacite plug making up part of the Pico El Laco central peak. We have not identified such a unit during our field studies, but remote infrared images indicate an area of distinct absorption on the NE-flank of the central peak (teal streak in Fig. 1a) with a spectral signature that correlates with silicic ignimbrites in the area. Much of the Si-rich endmember of unmixing may well have remained buried due to its comparatively high viscosity linked to its elevated Si-content and crystallinity. Evidence of Fe-rich magma intermingling with dacite magma preserved in a series of intrusive bodies in the similar MtAp deposits of Marcona, Peru^52^ show that unmixed magmas may remain in the subsurface.

A third open question is in what respect El Laco is special compared to other volcanoes in the region. Surrounding volcanoes of the Central Volcanic Zone show similar bulk lava compositions^9^. Near El Laco some smaller scale MtAp-type prospects such as Incahuasi and Cerro Imán are hosted by altered volcanics similar in infrared signature to El Laco (Fig. 1a). Tuyajito volcano some 10 km west of El Laco presents a conspicuos steam-heated alteration zone near its summit (Fig. 1a) similar to the Pasos Blancos area on El Laco known to overlay magnetite-rich dykes and breccias^25^. It is possible, therefore, that magma unmixing has occurred in other locations in the region but is not as conspicuously preserved or exposed at the surface as at El Laco. Of particular interest is Láscar, the currently most active volcano of the region. If it were true that magma unmixing is not limited to El Laco, then that volcano offers the tantalising prospect of detecting signatures of subsurface unmixing on a still active system, for example, by remote detection of gravity and magnetic anomalies linked to subsurface magnetite bodies, or seismic detection of magnetite dyke emplacement.

### Broader implications

Whereas we have tested this genetic model specifically for the case of El Laco, comparable evidence of intrusive and extrusive emplacement of Fe-rich orthomagmatic liquid into fractured host rock, association with cogenetic intermediate to felsic igneous rocks, and striking similarities in trace and stable isotope geochemistry between MtAp deposits^53^ indicate that our genetic model may apply to MtAp deposits globally. For example, dominantly explosive MtAp-deposits are reported at Cerro del Mercado, Mexico^54^, whereas ore bodies at the type-locality for MtAp-deposits at Kiruna, Sweden^55^ as well as the deposits at Marcona, Peru^52^, have been interpreted as intrusive structures. Magnetic anomalies at El Laco have also been interpreted as evidence of a further intrusive magnetite body beneath^56^. While vapour-driven ascent along fracture zones is a viable process in shallow subvolcanic environments such as at El Laco and Cerro del Mercado and other caldera-related MtAp systems, intrusive emplacement by pressurisation of an unmixing magma body by collapse- or regional tectonics-related faulting in transpressional/transtensional environments may still be a relevant process in deeper environments such as in the nearby Coastal Cordillera of Chile^57^. Whereas some degree of Fe-Si liquid immiscibility may be more ubiquitous, the evidence suggests that contamination by Fe-, P-, and/or S-rich basement lithologies and moderate extension (or trans-tension) around a cooling and deflating subvolcanic magma body provided an ideal environment for massive ore extrusion at El Laco.

Further experimental work into the Fe-Si liquid immiscibility will help to better constrain petrological, geochemical, and alteration signatures in Si-rich volcanic or plutonic rocks that would suggest the loss of an Fe-rich melt and its potential nearby emplacement. The denser melt may well have remained buried but should be detectable by magnetic, resistivity, and/or gravity surveys, or by related alteration signatures of overlaying rocks. The potentially efficient separation of an Fe-rich melt from a partially crystalline silicate magma body is not yet fully understood but may have implications for arc magma differentiation and continental crust formation. Due to the high density and low viscosity of the Fe-rich melt, magma unmixing can provide a mechanism for differentiating an andesite towards rhyolitic composition at relatively rapid rates compared to both crystal-settling in liquid-dominated magma^39^, as well as compaction in solid-dominated mush bodies^58^.

Finally, our findings may have important applications in planetary research. Crustal rocks on Mars exhibit significantly higher Fe-, and moderately elevated P-contents compared to Earth^59^. While the volatile budget and oxidation state remain debated, its crustal composition would suggest that Fe-Si magma unmixing is more likely to occur on Mars than on Earth. The consequent potential to facilitate silicate differentiation and generate ore bodies should be of great interest. It is possible that a candidate for such a deposit has already been identified at Terra Meridiani^60^. Moreover, it has been noted^61^ that the pronounced crustal magnetic anomalies found in parts of the Southern Highlands on Mars^62^ exhibit similar magnetic anomalies to the El Laco deposits^56^. Perhaps El Laco’s exotic ore forming melts are more common on other planets than on Earth.

## Methods

### Melt inclusions

Three thin sections prepared from andesite samples LCO-1, LCO-9, and LAC-AND^15^ containing immiscible melt inclusions were examined using an Olympus BX-50 transmitted light microscope and a JEOL-JSM 7100F field emission gun scanning electron microscope (FEG-SEM) with back-scattered electron (BSE) imaging capabilities at the Memorial University of Newfoundland. BSE images were collected under a 15 kV accelerating voltage.

### Fe-Si melt immiscibility model

The thermodynamic equilibrium calculator alphaMELTS^33,35^ detects liquid immiscibility by analysing the liquid free energy surface^36^. The model was not specifically calibrated to this compositional space. We compose a range of starting compositions from eight major oxides (Si, Ti, Al, Fe, Mg, Ca, Na, K), with added minor oxides of P and H to model El Laco andesite^9^. We partition Fe between ferric and ferrous oxides to control initial oxygen fugacity to 1.5–3 orders of magnitude above the quartz-fayalite-magnetite (QFM), a reasonable fO2 range for moderately oxidized arc magmas and similar to the experiments of refs. 18 and 19.

For each starting composition, we calculate phase equilibria at 10 ^∘^C intervals along an isobaric cooling path at a fixed pressure of 125 MPa (5 km depth) and from near the liquidus at 1050 to 850 °C, still well above the rhyolite eutectic. During the calculations, the oxygen fugacity is left unconstrained and generally decreases with cooling. We use averages of El Laco andesites as starting compositions (overall average, average of five highest-Si, and five lowest-Si samples) and add various amounts of H2O (1, 3, 5 wt%). All starting compositions are listed in Supplementary Table 1. The modelled phase assemblages and liquid compositions along the cooling path are shown in Supplementary Figs. 3–5. All results are reported after summing iron oxides to equivalent FeO_tot_, and normalising to unity sum over all oxides including phosphate and water. We also reproduce the experiments^18^, except for ones with added sulfur. The reproduced experimental results are shown in the background of our model results.

The MELTS algorithm detects Fe–Si liquid immiscibility for all tested compositions. For some model compositions, the algorithm identifies minor fractions of a third, Ca-P-rich liquid close to apatite stoichiometry. Although El Laco is relatively poor in phosphate minerals, some euhedral apatites are found in cavities near Crystales Grandes (name derives from the large apatite crystals found there)^9^. Moreover, Fe-phosphate alteration minerals (e.g., destinesite) found in Fe-rich tephra layers near Laco Sur^63^ may indicate that a third, P-rich liquid has been present during extrusion. The authors are not aware of experimental evidence of a third liquid. For ease of comparison, and to avoid overinterpreting the MELTS liquid immiscibility model, we report results after adding the latter two liquids up to a variably Fe- to Fe–Ca–P-rich liquid similar to those confirmed by experiment^18^.

### Melt viscometry

Superliquidus viscosities were determined using the concentric cylinder method. Raw samples were melted in thin-walled Pt_80_Rh_20_ crucibles to temperatures above their liquidus for several hours and then quenched by pouring from the crucible onto a steel plate (low viscosity melts) or cooling in the crucible (high viscosity melts) and drilled out using diamond coring tools. The viscometry samples so obtained were then remelted in Pt_80_Rh_20_ viscometry crucibles and loaded into a viscometry furnace where a Pt_80_Rh_20_ spindle was immersed into the melt sample at superliquidus conditions. Viscosity determinations were made in descending temperature steps and a final reoccupation of the highest temperature determination was obtained as a check against sample or instrument drift. Chemical analyses of samples were obtained by electron-probe micro-analysis post-viscometry. Sample analyses, viscosity measurments, and instrument calibration are reported in Supplementary Table 2.

The viscometry data is fitted to a temperature- and composition-dependent model to extrapolate values to the conditions relevant for subvolcanic conditions at El Laco. The fitted viscosity law, *η*(*T*, *c*_*j*_), as a function of temperature *T* and composition *c*_*j*_, is chosen of the form,

2 $$\eta (T,{c}_{j})={A}_{0}({c}_{j})\exp \left(\frac{{E}_{a}({c}_{j})}{RT}\right),$$

with *R* the universal gas constant, and the prefactor *A*_0_(*c*_*j*_) and activation energy *E*_*a*_(*c*_*j*_) two composition-dependent fitting parameters to be determined. The compositional variables *c*_*j*_ are the same as used in the ternary in Fig. 3 and include components of silica (*c*_Si_), Al-Na-K-oxides (*c*_Al_), and Fe-Ti-Mg-Ca-P-oxides (*c*_Fe_). The three components are found by summing up the oxide concentrations analysed from the experimental materials on which viscometry was performed and normalising to unity sum. Hence, only two of the three components are linearly independent. We chose the silica component as the dependent one and only include the latter two in the fitting procedure. First, the temperature-dependence of viscosity is fitted for each measured composition separately to determine best-fit values for the host andesite composition (*A*_0_ = 3.6744 × 10^−9^ Pas, *E*_*a*_ = 3.1815 × 10^5^ J/mol), and the Si-rich rhyolite (*A*_0_ = 2.2376 × 10^−9^ Pas, and *E*_*a*_ = 3.7267 × 10^5^ J/mol) and Fe-cpx compositions (*A*_0_ = 9.3725 × 10^−3^ Pas, *E*_*a*_ = 6.1011 × 10^4^ J/mol) from melt inclusions. The temperature-dependence of the magnetite ore melt cannot be fitted since only one data point was obtained of that composition. Using the best-fit values of the first step, the prefactor and activation energies are then each fitted as log-linear functions of composition,

3a $${A}_{0}({c}_{j})=\exp \left(B+C{c}_{{{{{{{{\rm{Fe}}}}}}}}}+D{c}_{{{{{{{{\rm{Al}}}}}}}}}\right),$$

3b $${E}_{a}({c}_{j})=\exp \left(E+F{c}_{{{{{{{{\rm{Fe}}}}}}}}}+G{c}_{{{{{{{{\rm{Al}}}}}}}}}\right),$$

with *B*, *C*, *D*, *E*, *F*, and *G* the fitting parameters. The fitted parameter values are *B* = −2.1207, *C* = −1.3624, *D* = −68.7967, *E* = 11.8739, *F* = −1.6950, and *G* = 3.9259. With these, *A*_0_ and *E*_*a*_ can be calculated for the magnetite-rich ore composition (*A*_0_ = 2.4607 × 10^−2^ Pas, *E*_*a*_ = 2.6871 × 10^4^ J/mol). The fitted viscosity values compared to the viscometry measurments are shown in Fig. 4. All fitting operations were achieved by the least-squares method in Matlab.

### Fe-rich melt separation model

We use the formalism of ref. 43 to analyse the characteristic scales of Fe-rich melt separation from the host magma. The analysis requires a phenomenological calibration of multi-phase transport coefficients based on pure-phase material properties as well as the connectivity of phase constituents at the micro-scale. For the former, we use the viscosity measurements detailed above as constraints (values used specified in the main text). For the latter, we rely on the insights taken from observations on melt inclusions (Fig. 3,^15^) and immiscibility experiments^19^, along with conceptual^40^, analogue^41^, and numerical modelling^42^ to inform a tentative phenomenological calibration of phase connectivity functions (see Supplementary Fig. 6). The solid phase disaggregates at *ϕ*^xtl^ ≈ 70% in the presence of wetting Si-rich melt, and at *ϕ*^xtl^ ≈ 50% where the non-wetting Fe-rich melt dominates. The Si-rich melt has a percolation threshold at *ϕ*^mSi^ ≈ 5%, whereas the Fe-rich melt has one at *ϕ*^mFe^ ≈ 10%. At 10% ≤ *ϕ*^mFe^ ≤ 20%, the connectivity of the Fe-rich melt steps up significantly as crystallinity increases above 50–60%. The calibration is similar to that proposed for the three-phase system of silicate crystals and melt (wetting fluid) with a magmatic volatile vapour (non-wetting fluid) in ref. 43 (see their Appendix Fig. A1). We use the calibrated coefficient model to calculate the characteristic speed of magma convection driven by the buoyancy contrast of a perturbation in Fe-rich melt fraction Δ*ϕ*^mFe^ = 1% of *ℓ*_0_ = 1 m size relative to the mixture density $\bar{\rho }$, and resisted by the effective mixture viscosity ${\bar{K}}_{v}$^43^,

4 $${u}_{0}=\frac{{{\Delta }}{\phi }^{{{{{{{{\rm{mFe}}}}}}}}}({\rho }^{{{{{{{{\rm{mFe}}}}}}}}}-\bar{\rho }){g}_{0}{\ell }_{0}^{2}}{{\bar{K}}_{v}}\,,$$

with *g*_0_ gravity. We further quantify the characteristic speed of Fe-rich melt segregation driven by the phase buoyancy contrast and resisted by the phase segregation coefficient ${{\phi }^{{{{{{{{\rm{mFe}}}}}}}}}}^{2}/{C}_{v}^{{{{{{{{\rm{mFe}}}}}}}}}$^43^,

5 $${w}_{0}=\frac{({\rho }^{{{{{{{{\rm{mFe}}}}}}}}}-\bar{\rho }){g}_{0}{{\phi }^{{{{{{{{\rm{mFe}}}}}}}}}}^{2}}{{C}_{v}^{{{{{{{{\rm{mFe}}}}}}}}}}\,.$$

The segregation coefficient is a generalisation of the Darcy percolation coefficient and depends on phase connectivity such that segregation mobility steps up where the Fe-rich melt transitions from disconnected droplets suspended in Si-rich melt to interconnected channels in crystal-rich mush. Lastly, we calculate the segregation-compaction length relating to the Fe-rich melt segregating from a compacting mixture of Si-rich melt and crystals,

6 $${\delta }_{0}=\max \left[\sqrt{\frac{{{\phi }^{{{{{{{{\rm{mFe}}}}}}}}}}^{2}{{\phi }^{{{{{{{{\rm{xtl}}}}}}}}}}^{2}}{{{C}_{v}^{{{{{{{{\rm{mFe}}}}}}}}}}{C}_{\phi }^{{{{{{{{\rm{xtl}}}}}}}}}}},\sqrt{\frac{{{\phi }^{{{{{{{{\rm{mFe}}}}}}}}}}^{2}{{\phi }^{{{{{{{{\rm{mSi}}}}}}}}}}^{2}}{{{C}_{v}^{{{{{{{{\rm{mFe}}}}}}}}}}{C}_{\phi }^{{{{{{{{\rm{mSi}}}}}}}}}}}\right]\,,$$

with ${{\phi }^{{{{{{{{\rm{xtl}}}}}}}}}}^{2}/{C}_{\phi }^{{{{{{{{\rm{xtl}}}}}}}}}$ and ${{\phi }^{{{{{{{{\rm{mSi}}}}}}}}}}^{2}/{C}_{\phi }^{{{{{{{{\rm{mSi}}}}}}}}}$ the compaction coefficients^43^ of the crystal and Si-rich melt phases, respectively.

### Volcano deformation model

We use a two-dimensional, finite-element model based on the methods of refs. 45 and 46 solving the Stokes equations for a visco-elastic/brittle-plastic material forced by topographic loads, far-field tectonic deformation rates, and a subvolcanic volume contraction/expansion source. The governing equations are,

7a $${{{{{{{\boldsymbol{\nabla }}}}}}}}P={{{{{{{\boldsymbol{\nabla }}}}}}}}\cdot {{{{{{{\boldsymbol{\tau }}}}}}}}+\rho {{{{{{{\bf{g}}}}}}}}\,,$$

7b $${{{{{{{\boldsymbol{\nabla }}}}}}}}\cdot {{{{{{{\bf{v}}}}}}}}=A \dot{\upsilon }\,,$$

where *P* is the Stokes pressure, **v** the Stokes velocity, *ρ* the density, **g** the gravity vector, and ***∇*** the spatial gradient operator. On the right-hand side of (7b) we impose a de-/inflation rate (volumetric strain rate) of amplitude $\dot{\upsilon }$ and spatial extent given by a dimensionless Gaussian shape function, *A*. The shear stress tensor, ***τ***, is given by a Maxwell visco-elastic flow law (for details see refs. 45, 46),

8 $${{{{{{{\boldsymbol{\tau }}}}}}}}={\eta }_{ve}{{{{{{{\bf{D}}}}}}}}({{{{{{{\bf{v}}}}}}}})+{\chi }_{ve}{\tilde{{{{{{{{\boldsymbol{\tau }}}}}}}}}}^{o}\,,$$

as a function of the deviatoric strain rate tensor tensor, **D**(**v**), and the stress state, ${\tilde{{{{{{{{\boldsymbol{\tau }}}}}}}}}}^{o}$, a discrete time step Δ*t* prior. The rheology is governed by the rock visco-elasticity, *η*_*ve*_, and the visco-elastic stress parameter, *χ*_*ve*_,

9a $${\eta }_{ve}={\left(\frac{1}{\eta }+\frac{1}{G{{\Delta }}t}\right)}^{-1}\,,$$

9b $${\chi}_{ve}={\left(1+\frac{G{{\Delta }}t}{\eta }\right)}^{-1}\,,$$

which depend on the shear viscosity, *η*, and shear modulus, *G*, of the deforming rock. Elastic strain is included as a time step-dependent, pseudo-viscous component of strain rate while not resolving elastic waves on a seismic time scale.

The brittle-plastic component of rock deformation is imposed in the form of a stress limiter on the shear stress magnitude, $\tau=\sqrt{{{{{{{{\boldsymbol{\tau }}}}}}}}:{{{{{{{\boldsymbol{\tau }}}}}}}}/2}$. Shear stress is limited to remain below a Mohr-Coulomb yield stress,

10 $${\tau }_{y}={C}_{0}+{\mu }_{0}P\,,$$

a function of the rock cohesion, *C*_0_, and the friction coefficient, *μ*_0_. The weakening effect of elevated temperature and partial melt around a subvolcanic magma body is represented by a parameterised rheological weakening, *η* = *η*_0_*Ar*_*η*_ where, *η*_0_ is the background rock viscosity, *r*_*η*_ is a dimensionless weakening factor, and *A* is the same Gaussian shape function used for the de-/inflation source in (7b).

The boundary conditions are stress-free (∂**v**/∂**n** = 0; *P* = 0) along the surface, shear stress-free along the sides (∂*w*/∂*x* = 0; *u* = *u*_*BC*_; ∂*P*/∂*x* = 0) and no-slip along the base of the domain (*u* = *u*_*BC*_; *w* = *w*_*BC*_; ∂*P*/∂*z* = 0). Boundary-normal velocity components on the sides and both components along the base are set to a pure shear field, [*u*_*BC*_, *w*_*BC*_], to allow for tectonic extension or compression to be imposed. The initial geometry of the 2-D vertical cross-section domain has a Gaussian topographic peak representing the volcanic edifice and comprises two subsurface layers of distinct deformational properties. The top layer is set to a higher viscosity reflecting a geological setting of more competent, young volcanic units overlaying a weaker metasedimentary basement. The shear modulus and yield parameters are held constant throughout the domain. On the subsurface layer boundary and centred beneath the edifice peak, we add the subvolcanic magma reservoir marked by the reduced viscosity and adjustable volume de-/inflation rate as described above. We assume horizontal symmetry across an axis extending vertically beneath the volcanic peak and hence only perform calculations on one half of the domain.

The model is controlled by two dimensionless groups of parameters characterising the stress induced by far-field tectonic deformation (imposed as boundary conditions) and by volumetric deformation of the magma body (imposed as volume source) relative to the characteristic yield stress of the volcanic edifice,

11a $${{{{{{{\mathcal{S}}}}}}}}=\frac{{\dot{\varepsilon }}_{0}{\eta }_{0}}{{C}_{0}+{\mu }_{0}{P}_{0}}\,,$$

11b $${{{{{{{\mathcal{T}}}}}}}}=\frac{{\dot{\upsilon }}_{0}{\eta }_{0}\sqrt{{r}_{\eta }}}{{C}_{0}+{\mu }_{0}{P}_{0}}\,.$$

For the chosen yield parameters (*C*_0_ = 10 MPa, *μ*_0_ = 0.3), lithostatic pressure at the depth of the magma body (*P*_0_ = 125 MPa), rock viscosity of the edifice (*η*_0_ = 10^23^ Pas), and magma weakening factor (*r*_*η*_ = 10^−4^), a source number of ${{{{{{{\mathcal{S}}}}}}}}=\pm 1$ corresponds to an in-/deflation rate of ${\dot{\upsilon }}_{0}=\pm 4.75\times 1{0}^{-14}$ s^−1^, and a tectonic number of ${{{{{{{\mathcal{T}}}}}}}}=\pm 1$, to an extension/compression rate of ${\dot{\varepsilon }}_{0}=\pm 4.75 \times 10^{-16}$ s^−1^. We test all combinations of the two governing numbers (${{{{{{{\mathcal{S}}}}}}}},\,{{{{{{{\mathcal{T}}}}}}}}$) for values of ±[0, 1, 2, 4]. In addition, we take the parameter combination identified as best fit (${{{{{{{\mathcal{S}}}}}}}}={{{{{{{\mathcal{T}}}}}}}}=-1$) and test its robustness on a range of magma body geometries and depths, and edifice topographies. Lastly, we perform resolution tests to demonstrate model convergence when refining spatial and temporal resolution. Results of all parameter variations tested are given in Supplementary Figs. 7–9.

To compare the best-fit volume deflation rate to the scenario of ore liquid generation in a cooling magma reservoir, we consider the thermal expansivity, 1/*VdV*/*dT* (*V* the volume, and T the temperature), of the bulk phase assemblage given by the alphaMELTS model. The thermodynamically predicted deflation rate for the tested temperature range can be determined by the relation,

12 $$\dot{\upsilon }=\frac{1}{V}\frac{dV}{dT}\frac{dT}{dt}\,,$$

where the temperature rate, *dT*/*dt*, is assumed to be that of diffusive cooling of a crustal magma body, which scales as,

13 $$\frac{dT}{dt} \sim \frac{{\kappa }_{0}{{\Delta }}{T}_{0}}{{R}_{0}^{2}}\,,$$

with *κ*_0_ the thermal diffusivity, Δ*T*_0_ the temperature difference between magma body and country rock, and *R*_0_ the characteristic size of the thermal aureole. The latter depends on the age and thermal maturity of the crustal magmatic system, which are not well constrained at El Laco. We assume that *R*_0_ will likely be more than 1 km but less than the depth of the magma reservoir (4–6 km). With Δ*T*_0_ = 800 °C (liquidus of 1100 °C, crustal temperature of 300 °C), *κ*_0_ = 10^−6^  m^2^/s (typical for silicates), and 1 ≤ *R*_0_ ≤ 4 km, we estimate cooling rates of order 10^−10^ ≤ *dT*/*dt* ≤ 10^−9∘^C/s. For El Laco andesite undergoing cooling, crystallisation, and liquid immiscibility at a temperature of 1050–850^∘^C, and *P* = 125 MPa, alphaMELTS calculates a thermal expansivity of order $1{0}^{-{4}^{\circ }}$C^−1^. With the cooling rate estimated above, a reasonable range of deflation rates hence is $1{0}^{-14}\,\le \,{\dot{\upsilon }}_{0}\,\le \,1{0}^{-13}$ s^−1^, which brackets the best-fit deflation rate identified in the volcano deformation model above.

### Fracture-bound bubbly flow model

To analyse the physical scales of bubbly fracture flow, we formulate a simplified model equation based on the multi-phase reactive transport theory of ref. 43. We assume the limit of an incompressible liquid bearing small, well-distributed, and fully entrained bubbles of compressible vapour. We further assume that flow is along a straight and smooth fracture with rigid and impermeable walls, and with an opening much smaller than its length, and hence apply the lubrication limit to the along-fracture flow. That is, shear stresses along the wall are assumed to balance the pressure drop that drives the flow. Consequently, the flow assumes a parabolic profile across the fracture opening following the solution of plane-Poiseuille flow. The unknown variable of interest is the magnitude of the along-fracture ascent speed, *w* (see Supplementary Fig. 10).

Using conservation of mass in the bubbly liquid (eq. (38b) in ref. 43), the along-fracture gradient of the ascent speed is given by,

14 $$\frac{\partial w}{\partial z}=-\frac{\phi }{{\rho }^{g}}\frac{D{\rho }^{g}}{Dt}+{{{\Gamma }}}_{\rho }\left(\frac{1}{{\rho }^{g}}-\frac{1}{{\rho }^{\ell }}\right)\,,$$

where *ϕ* is the volume fraction of bubbles entrained in the flow, *ρ*^*g*^ and *ρℓ* are the gas and liquid densities, respectively, Γ_*ρ*_ the mass transfer rate from liquid to gas, or vapour exsolution rate, and $\frac{\phi }{{\rho }^{g}}\frac{D{\rho }^{g}}{Dt}$ the rate of gas density change in a material reference frame moving with $w$, or the vapour expansion rate. The vertical coordinate $z$ is aligned with the fracture and increases upwards from the origin at the fracture inlet; the fracture is inclined with respect to gravity, *g*, by an angle *α* (see Supplementary Fig. 10).

For simplicity, we assume that both the vapour exsolution and expansion rates are linear functions of pressure, and that the decompression rate upon ascent is dominated by flow against the hydrostatic pressure gradient,

15a $${{{\Gamma }}}_{\rho }=\phi (1-\phi )\gamma {\rho }^{\ell }g\cos (\alpha )w\,,$$

15b $$\frac{\phi }{{\rho }^{g}}\frac{D{\rho }^{g}}{Dt}=-\phi (1-\phi )\beta {\rho }^{\ell }g\cos (\alpha )w\,.$$

The two control parameters are the gas compressibility, *β* [1/Pa], and the exsolution productivity, *γ* [kg/m^3^/Pa]. Substituting back into (14) and grouping of terms yields,

16 $$\frac{\partial w}{\partial z}=\phi (1-\phi ){\rho }^{\ell }g\cos (\alpha )\left(\beta+\frac{\gamma }{{\rho }^{g}}\right)w\,,$$

To arrive at (16) we have used *ρ*^*g*^ ≪ *ρ*^*ℓ*^ to simplify (1/*ρ*^*g*^ − 1/*ρ*^*ℓ*^ ≈ 1/*ρ*^*g*^. Equation (16) states that the along-fracture gradient of ascent speed is a function of $w$ itself, hence indicating an exponential solution. By integrating (16) along the fracture while holding all other variables constant we find a solution for *w* of the form,

17 $$w={w}_{{{{{{{{\rm{in}}}}}}}}}\exp (z/\lambda )\,,$$

with the prefactor determined by the boundary condition, *w*(*z* = 0) = *w*_in_, i.e., the speed of liquid injection at the base of the fracture; we determine *w*_in_ from a plane-Poiseuille flow law driven by the collapse-induced magma chamber pressure jump, Δ*P*_0_, over the horizontal extent of the inclined fracture, ${L}_{0}\sin (\alpha )$,

18 $${w}_{{{{{{{{\rm{in}}}}}}}}}=\frac{{{\Delta }}{P}_{0}{H}_{0}^{2}}{2{\eta }_{0}^{\ell }{L}_{0}\sin (\alpha )}\,.$$

*L*_0_ and *H*_0_ are the fracture length and opening, respectively, and ${\eta }_{0}^{\ell }$ the effective viscosity of the bubbly liquid. The *e*-fold growth length of the exponential law in (17) is a function of the model parameters,

19 $$\lambda={\left[\phi (1-\phi ){\rho }^{\ell }g\cos (\alpha )\left(\beta+\frac{\gamma }{{\rho }^{g}}\right)\right]}^{-1}\,.$$

We define a non-dimensional number that characterises the exponential growth potential of the ascent speed,

20 $${{\Lambda }}={L}_{0}/\lambda \,.$$

If Λ ≤ 1 then the ascent speed will not grow much above the initial injection speed; if Λ ≥ 1, however, ascent speed will tend to grow exponentially along the fracture.

In Supplementary Table 3, we provide a summary of what we consider appropriate ranges for the physical parameters in this problem and use these to analyse the scales pertaining to ore liquid extrusion. We find that the dimensionless growth number for the ascent speed is typically larger than unity and can be as high as ~100.

## Supplementary information

Supplementary Information

## Data Availability

The authors declare that the data generated or analysed during this study are included in this published article and its Supplementary Information file. Raw viscometry measurements along with instrument calibration data are given in Supplementary Table 2. Raw output data produced with the alphaMELTS model are available for download from the Zenodo code repository 10.5281/zenodo.6982625^64^; input compositions are given in Supplementary Table 1, and processed output data are presented in Supplementary Figs. 3–5. Processed output data produced with the volcano deformation model are presented in Supplementary Figs. 6–8; raw output data can be reproduced with the code and input files provided on the Zenodo repository^64^ or are available from the corresponding author upon request.

## Code availability

The alphaMELTS code (v 1.9) is freely available for download from the authors’ website https://magmasource.caltech.edu/alphamelts/^65^. The custom-built codes and input files to reproduce the reconstructed andesite composition by least-squares fit, the model fit to viscometry data, the coefficient calibration and scaling analysis for Fe-rich melt segregation, and the volcano deformation model results are available for download from an open repository on Zenodo 10.5281/zenodo.6982625^64^.
