# Supplementary material for: Genetic model of the El Laco magnetite-apatite deposits by extrusion of iron-rich melt
Source: Nat Commun. 2022 Oct 17;13:6114. doi: 10.1038/s41467-022-33302-z (PMC9576724; doi:10.1038/s41467-022-33302-z)
Supplement: Supplementary file 1 — Supplementary Information [file 41467_2022_33302_MOESM1_ESM.pdf]

Supplementary Information to article

A genetic model of the magnetite-apatite deposits on El Laco volcano by extrusion of iron-rich melt

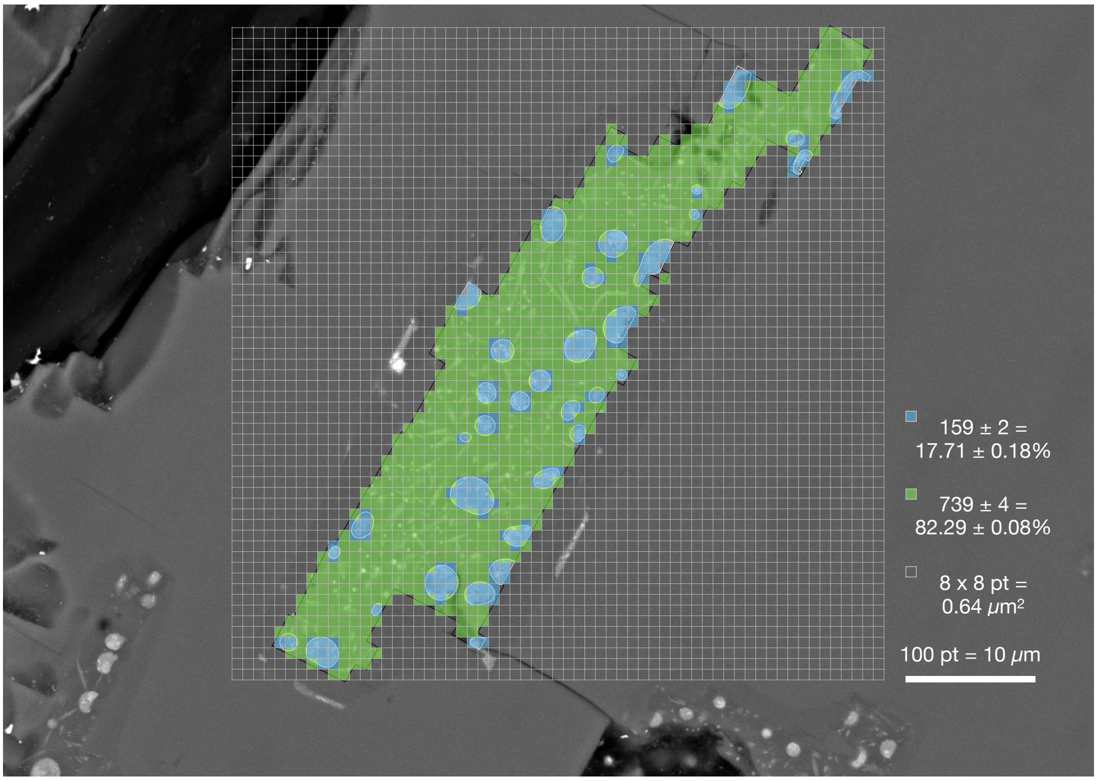

Supplementary Figure 1: Back-scattered electron microscopy image of melt inclusion in host andesite. Point-count for estimating modal abundances of Fe-rich blebs and Si-rich matrix phases; squares correspond to 0.8×0.8 μm. Point-count returns $17.71 \pm 0.18\%$ Fe-rich to $82.29 \pm 0.08\%$ Si-rich phases.

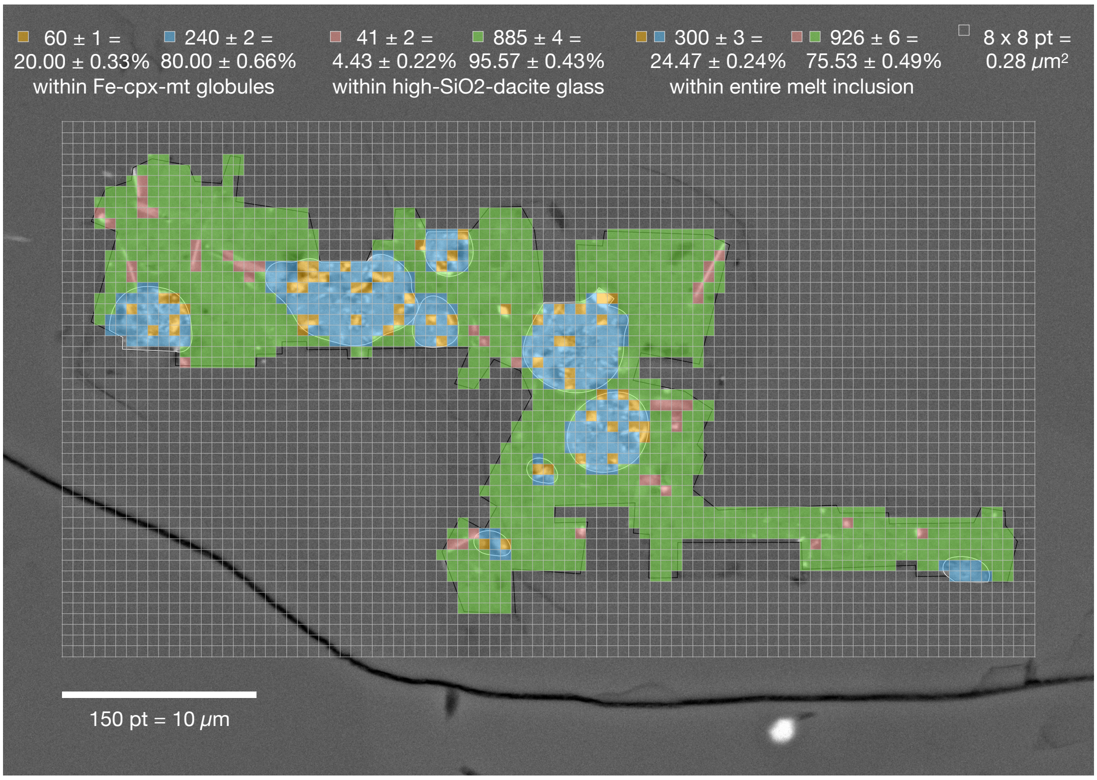

Supplementary Figure 2: Back-scattered electron microscopy image of melt inclusion in host andesite. Point-count for estimating modal abundances of Fe-clinopyroxene and magnetite in Fe-rich blebs and rhyolite glass matrix phases; squares correspond to 0.53×0.53 μm. Point-count returns $19.40 \pm 0.27\%$ magnetite to $80.60 \pm 0.12\%$ Fe-cpx in blebs, and $24.39 \pm 0.18\%$ Fe-rich to $75.61 \pm 0.12\%$ Si-rich phases.

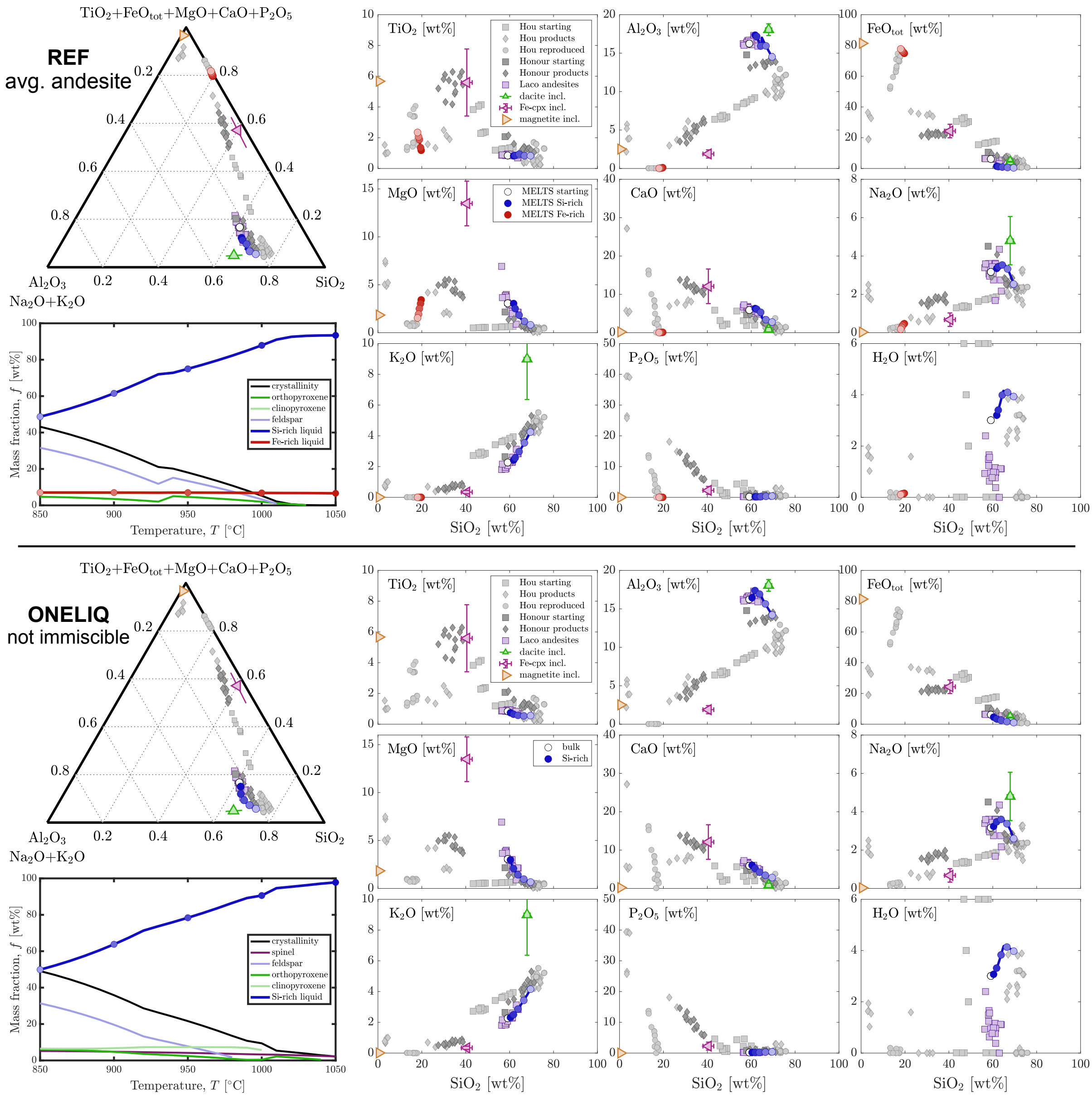

Supplementary Figure 3: Model results of thermodynamic equilibrium calculations for average El Laco andesite. With (REF) and without (ONELIQ) liquid immiscibility mode enabled. Error bars ±1 standard deviation.

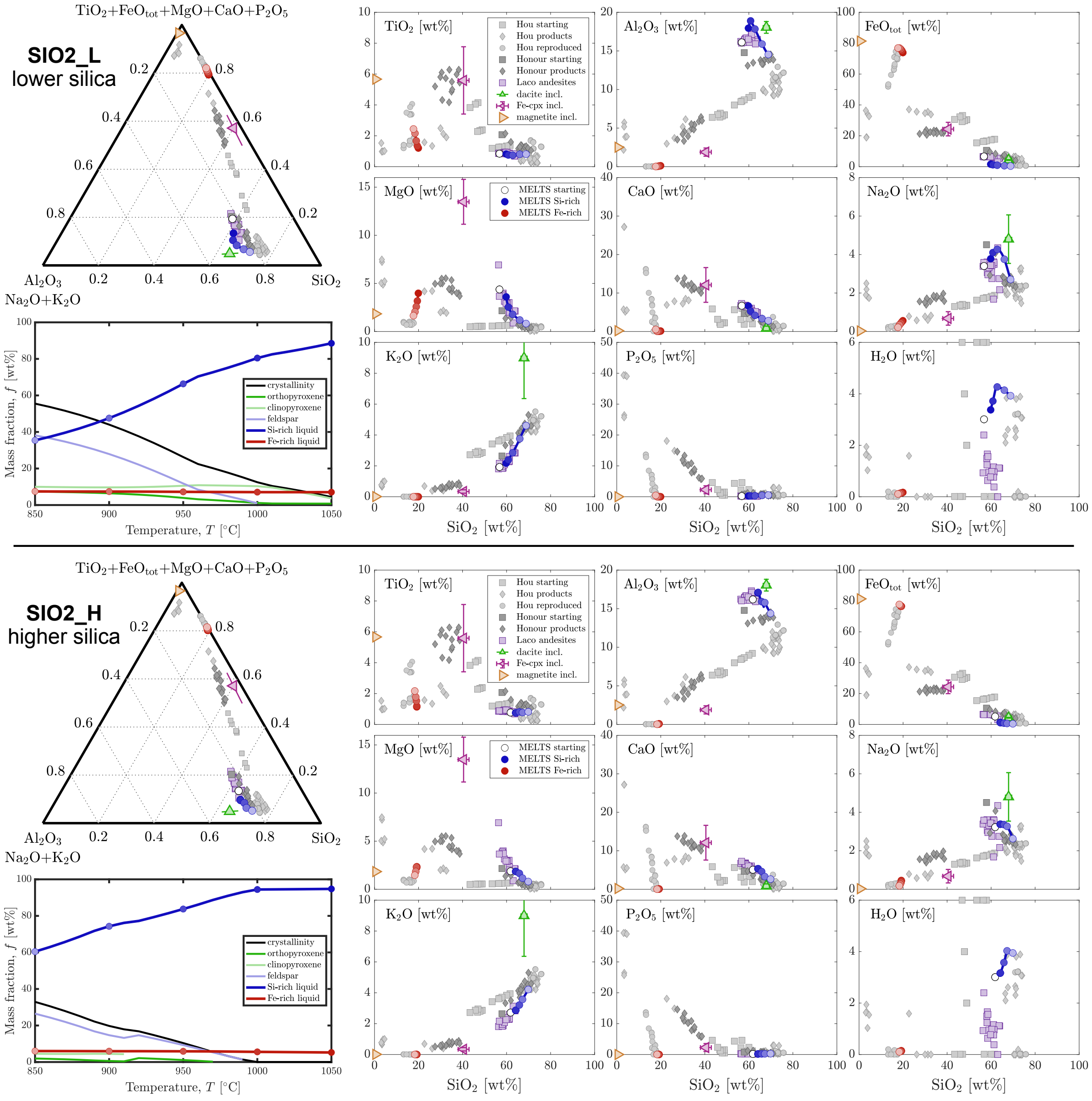

Supplementary Figure 4: Model results of thermodynamic equilibrium calculations for average El Laco andesite. Average of five lowest silica (SIO2_L) and five highest silica (SIO2_H) El Laco andesite compositions. Error bars ±1 standard deviation. Based on rock compositions in Fernando Tornos, Francisco Velasco, John M. Hanchar; The Magmatic to Magmatic-Hydrothermal Evolution of the El Laco Deposit (Chile) and Its Implications for the Genesis of Magnetite-Apatite Deposits. Economic Geology 2017;; 112 (7): 1595–1628. doi: https://doi.org/10.5382/econgeo.2017.4523

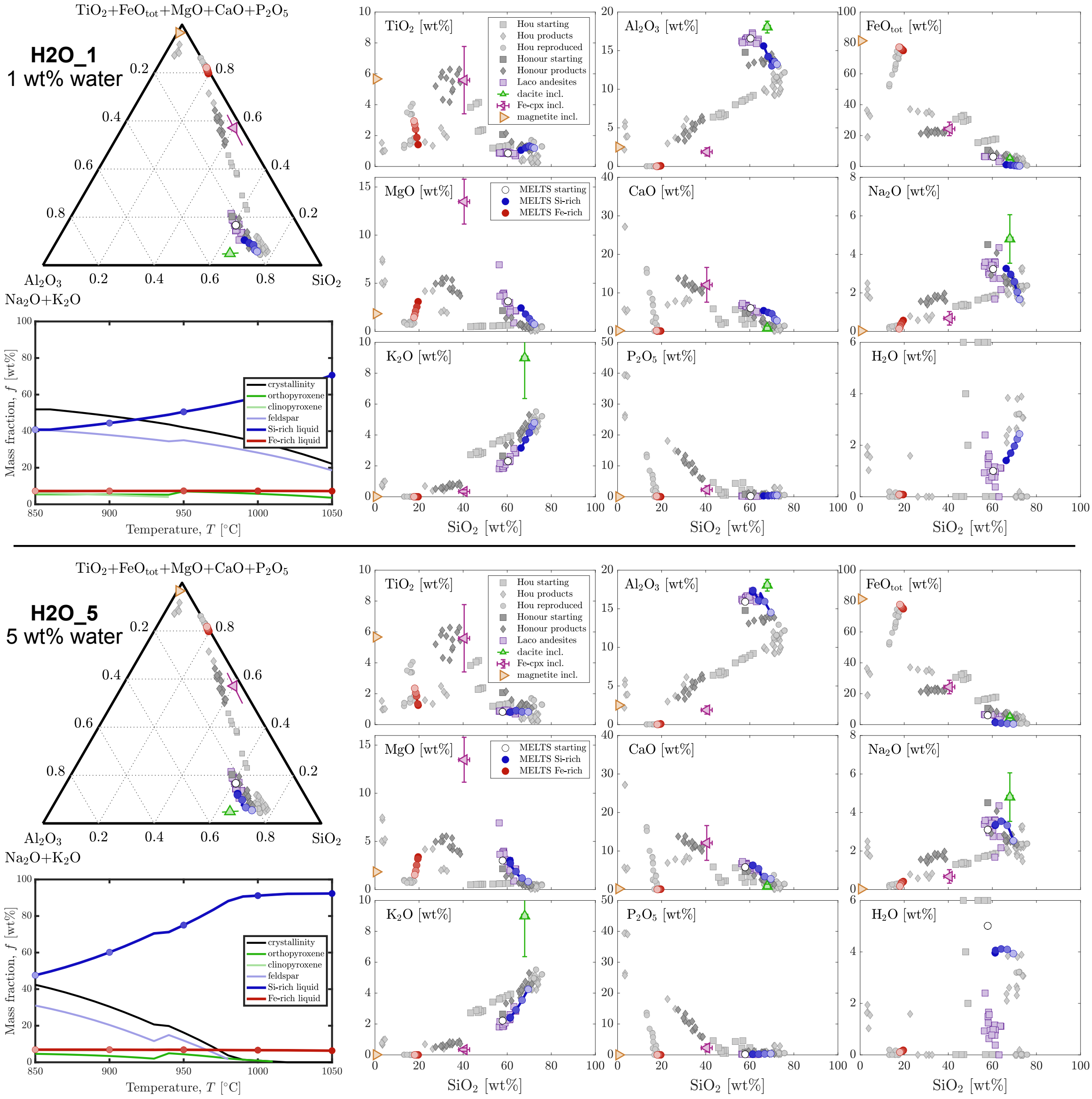

Supplementary Figure 5: Model results of thermodynamic calculations for average El Laco andesite. With 1 wt% (H2O_1) and 5 wt% (H2O_5) water added. Error bars ±1 standard deviation.

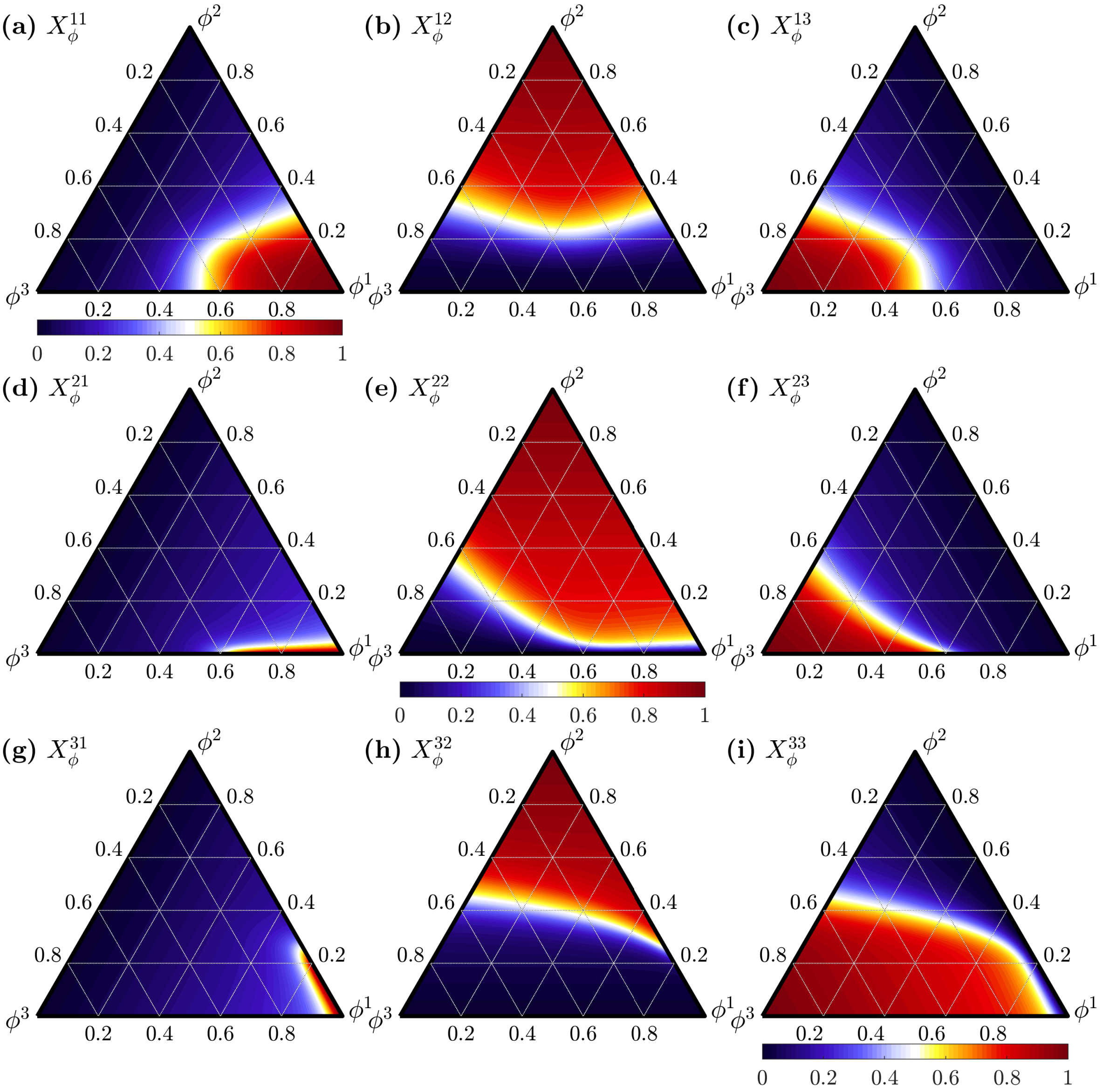

Supplementary Figure 6: Phenomenological connectivity functions for three-phase coefficient calibration used in the scaling analysis of ore melt separation. The end-members are the phenocryst ϕ^1, the Si-rich melt ϕ^2, and the Fe-rich melt ϕ^3 phases, connectivity functions are X_ϕ^{ij} between phases i, j. Panels show connectivity of phenocrysts with themselves (a), with Si-rich melt (b), and with Fe-rich melt (c); Si-rich melt with phenocrysts (d), with itself (e), and with Fe-rich melt (f); and Fe-rich melt with phenocrysts (g), with Si-rich melt (h), and with itself (i). Values of 1 correspond to fully interconnected, values of 0 to fully disconnected local-scale phase topologies.

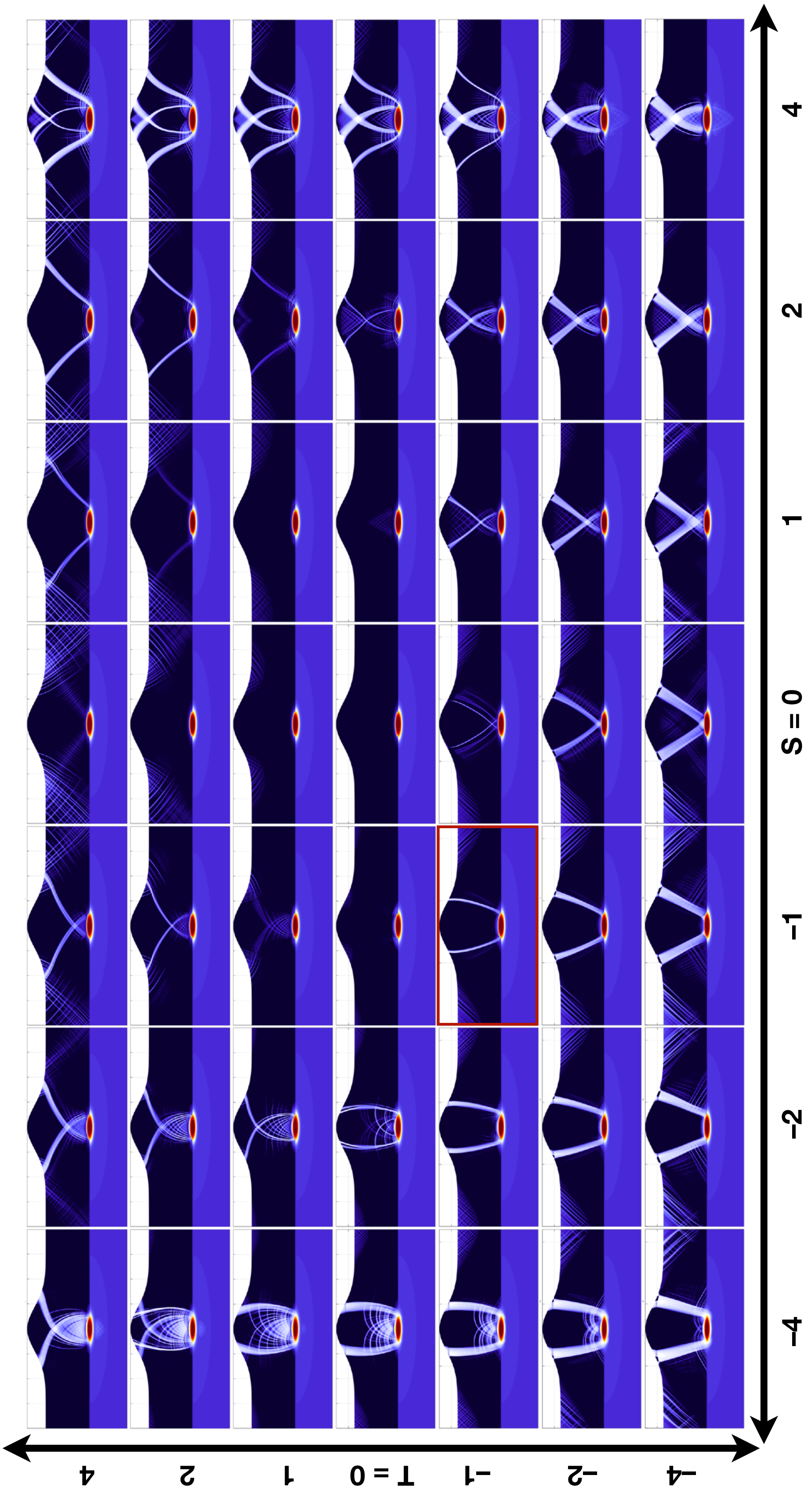

Supplementary Figure 7: Results of volcano deformation model with different forcing functions. Full range of parameters tested from deflating ($\mathcal{S} < 0$) to inflating ($\mathcal{S} > 0$) volume sources and extensional ($\mathcal{T} < 0$) and compressional ($\mathcal{T} > 0$) tectonic stress applied; best fit model marked in red; colour bar as in main text Fig. 5.

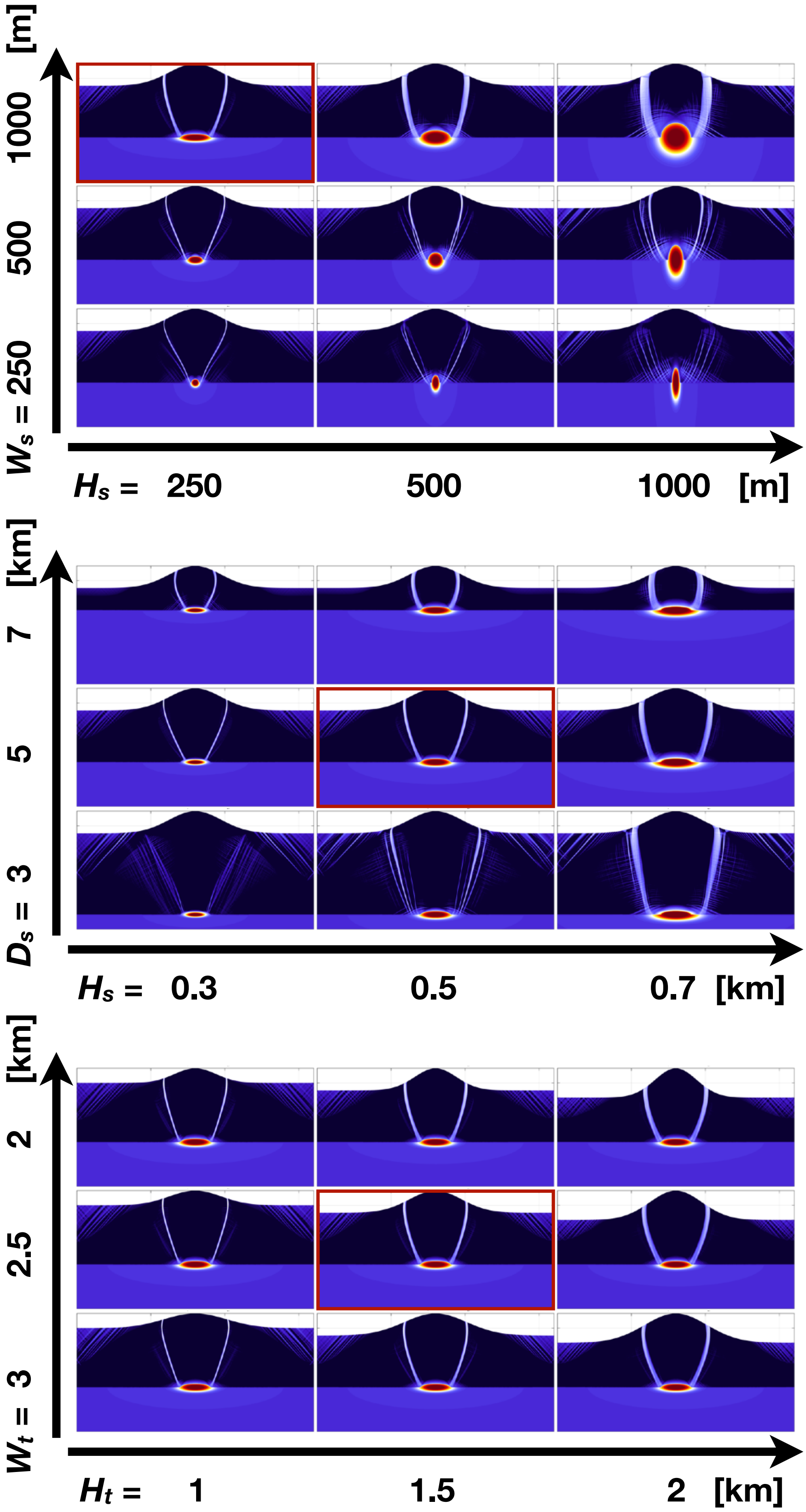

Supplementary Figure 8: Robustness of volcano deformation model results. Failure zone geometry is robust over wide range a of source geometries in width (W_s), height (H_s), and depth (D_s) and edifice topography width (W_t) and height (H_t); best fit model marked in red; colour bar as in main text Fig. 5.

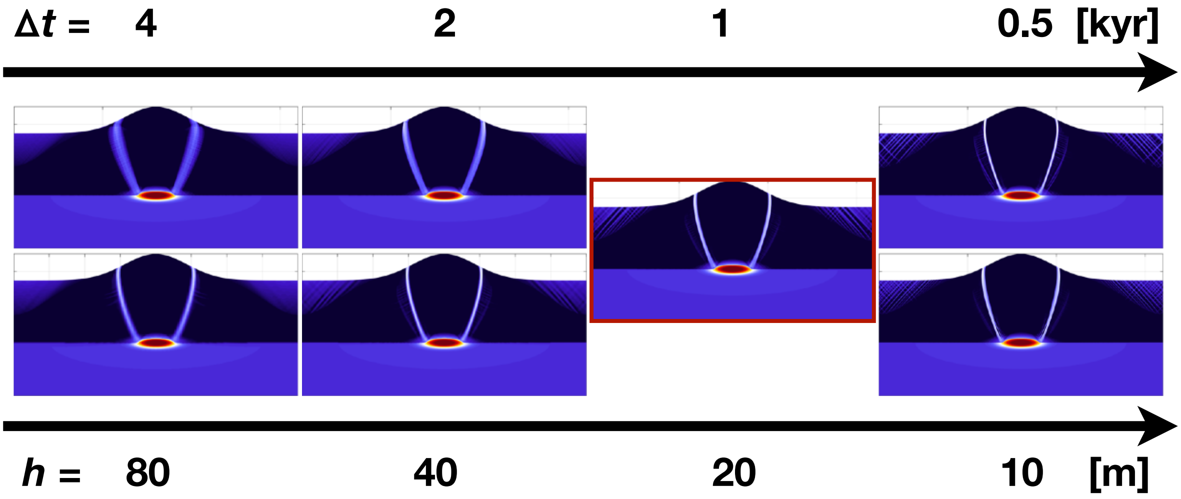

Supplementary Figure 9: Numerical convergence of volcano deformation model results. Resolution tests for time step (Δt) and grid step (h) size show convergence of model results to well-resolved failure pattern. Resolution used for best fit and other parameter tests above marked in red; colour bar as in main text Fig. 5.

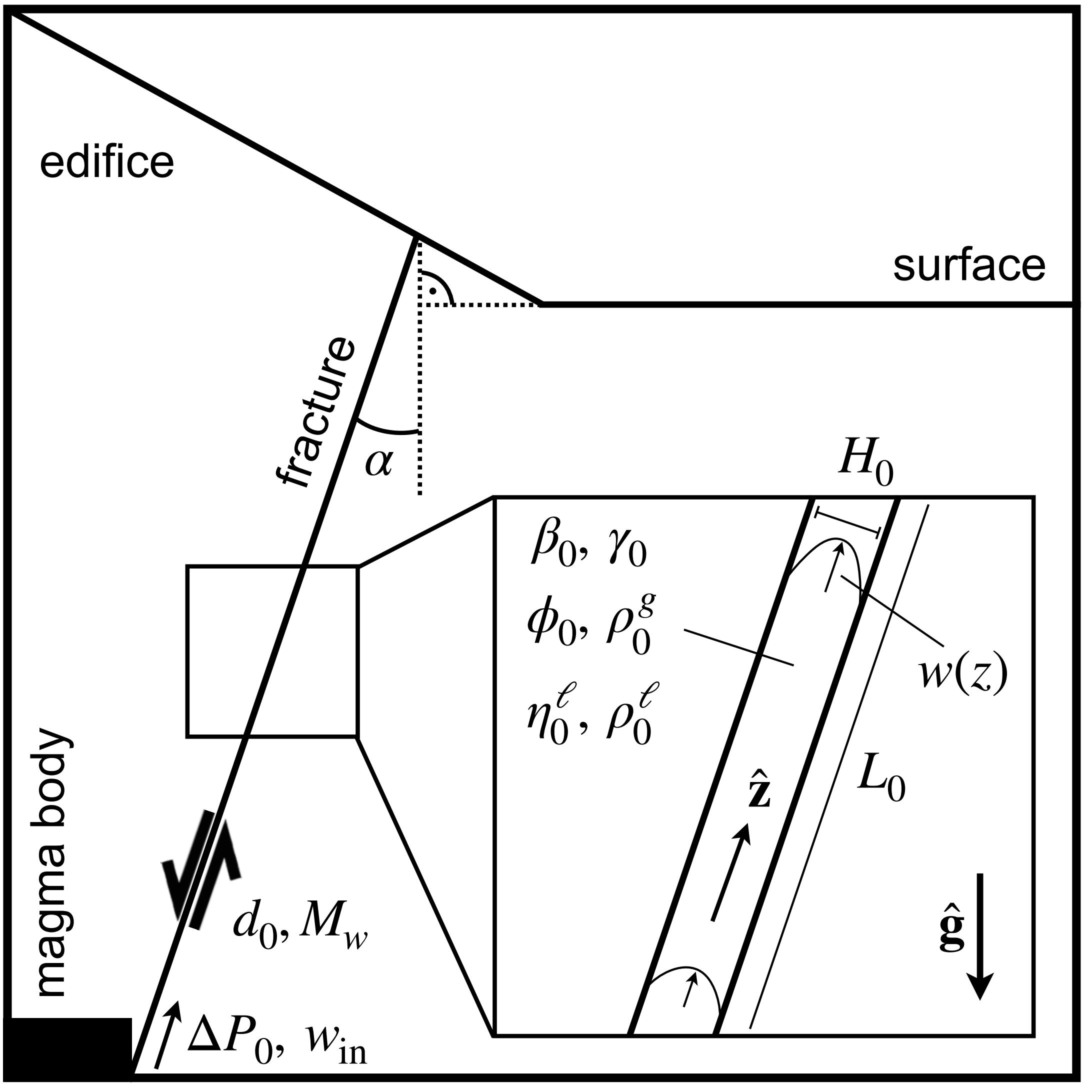

Supplementary Figure 10: Model sketch for collapse-driven injection and bubble exsolution-, and expansion-driven ascent of ore liquid. Mathematical symbols as defined in main text and Suppl. Table 3.

	SiO2	TiO2	Al2O3	FeOtot	MgO	CaO	Na2O	K2O	P2O5	H2O	SUM	REF
	**averages of FEG-EPMA compositions in plagioclase-hosted melt inclusions from El Laco andesite**											
**Inclusions high-SiO_2-dacite glass**	**67.94**	**0.25***	**18.05**	**4.32**	**0.06***	**0.80**	**4.80**	**8.99**	**0.20***	-	104.90	Pietruszka et al., 2022
**standard deviation**	1.40	0.02	0.76	0.22	0.01	0.60	1.26	2.63	0.02	-		
**Inclusions Fe-cpx in globules**	**40.48**	**5.59**	**1.89**	**24.35**	**13.48**	**12.10**	**0.68**	**0.34**	**2.26**	-	101.17	Pietruszka et al., 2022
**standard deviation**	2.46	2.18	0.53	4.39	2.33	4.51	0.35	0.11	1.29	-		
												*below detection limit
	**averages of published whole-rock and mineral compositions of samples taken at El Laco**										0.00	
**El Laco low-SiO_2 andesite**	57.50	0.86	16.31	6.73	4.43	6.78	3.45	1.95	0.22	1.54	99.78	Tornos et al., 2017
**El Laco mean andesite**	59.98	0.85	16.48	6.25	3.11	6.00	3.22	2.29	0.20	1.06	99.44	Tornos et al., 2017
**El Laco high-SiO_2 andesite**	62.64	0.78	16.41	5.21	1.90	5.14	3.27	2.75	0.17	0.71	98.97	Tornos et al., 2017
**El Laco incl. magnetite**	0.41	2.51	2.50	83.41	1.88	0.17	0.02	0.01	0.00	0.00	90.90	Velasco et al., 2016
**El Laco plagioclase**	54.25	0.04	29.57	0.66	0.02	10.96	4.19	0.45	0.00	0.00	100.15	Velasco et al., 2016
**El Laco orthopyroxene**	53.95	0.26	1.16	17.59	24.63	1.48	0.03	0.01	0.00	0.00	99.11	Velasco et al., 2016
**El Laco clinopyroxene**	51.94	0.63	2.38	8.64	14.15	21.26	0.37	0.01	0.00	0.00	99.38	Velasco et al., 2016
	**SiO2**	**TiO2**	**Al2O3**	**Fe2O3**	**FeO**	**MgO**	**CaO**	**Na2O**	**K2O**	**P2O5**	**H2O**	**SUM**
**MODELLED**	**composed from end-members above and used as starting compositions in alphaMELTS model runs**											
**REF / ONELIQ**	58.95	0.84	16.19	3.07	3.38	3.06	5.90	3.16	2.26	0.20	3.00	100.00
**REMIX**	58.01	0.79	16.18	3.02	3.33	3.30	5.94	2.94	3.23	0.26	3.00	100.00
**SIO2_L**	56.58	0.84	16.05	3.31	3.64	4.36	6.68	3.39	1.92	0.22	3.00	100.00
**SIO2_H**	61.67	0.77	16.16	2.56	2.82	1.87	5.06	3.22	2.71	0.17	3.00	100.00
**H2O_1**	60.17	0.85	16.53	3.13	3.45	3.12	6.02	3.23	2.30	0.20	1.00	100.00
**H2O_5**	57.74	0.82	15.86	3.01	3.31	3.00	5.78	3.10	2.21	0.19	5.00	100.00

1

Supplementary Table 1: Compositional averages taken from published analyses, and model compositions composed for our thermodynamic model calculations.

**Viscometry sample analyses (EPMA)**															
**Sample**	**SiO2**	**TiO2**	**Al2O3**	**Fe2O3**	**MgO**	**CaO**	**Na2O**	**K2O**	**P2O5**	**SUM**	**rting material**				
**Experiment host andesite**	64.23	0.87	17.25	3.51	1.00	5.61	4.52	3.14	0.13	100.26	sample ELL-18-01, Tornos et al., 2017				
**Experiment Si-rich matrix**	70.45	0.30	14.56	2.94	0.17	0.32	3.35	7.78	-	99.87	synthetic, after Pietruszka et al., 2022				
**Experiment Fe-cpx globules**	39.58	5.46	1.68	24.00	12.88	12.61	0.64	0.18	2.01	99.04	synthetic, after Pietruszka et al., 2022				
**Experiment magnetite ore**	0.07	0.39	0.30	99.35	0.77	0.04	-	-	-	100.91	sample ELL-13-05, Tornos et al., 2017				
**Viscometry measurements**				**host andesite**			**Si-rich rhyolite**			**Fe-cpx globules**			**magnetite ore**		
	T_{set}	T_{real}	$10^4/T_{real}$	**RPM**	τ	η	**RPM**	τ	η	**RPM**	τ	η	**RPM**	τ	η
	1650.00	1627.00	5.26				5.00	77.11	40.51				100.00	0.10	0.18
	1625.00	1603.00	5.33				2.50	52.53	54.24						
	1600.00	1579.00	5.40				2.50	70.64	73.15						
	1575.00	1555.00	5.47				2.50	95.81	99.44						
	1550.00	1531.00	5.54				1.20	64.13	136.20						
	1525.00	1507.00	5.62				1.20	88.67	188.78						
	1500.00	1483.00	5.69	20.00	84.93	12.00	0.60	63.43	266.62	40.00	2.49	0.62			
	1475.00	1459.00	5.77	10.00	56.98	15.61	0.60	89.63	377.73	40.00	3.00	0.65			
	1450.00	1435.00	5.85	10.00	76.32	20.68	0.30	65.21	546.40	40.00	3.52	0.69			
	1425.00	1411.00	5.94	5.00	52.11	27.57	0.30	94.93	801.64	40.00	4.04	0.72			
	1400.00	1387.00	6.02	5.00	70.77	37.23	0.20	93.08	1181.70	40.00	4.66	0.77			
	1375.00	1363.00	6.11	5.00	97.39	51.01	0.10	69.62	1763.62	40.00	5.42	0.82			
	1350.00	1339.00	6.20	2.50	68.72	71.14	0.05	53.04	2677.91	40.00	6.38	0.89			
	1325.00	1315.00	6.29	2.50	96.72	100.39	0.05	80.16	4117.14	40.00	7.54	0.97			
	1300.00	1291.00	6.39	1.20	67.24	142.86	0.05	122.68	6373.63						
	1275.00	1267.00	6.49	1.20	97.48	207.66									
	1250.00	1244.00	6.59	0.60	73.06	307.46									
	1225.00	1220.00	6.70	0.30	55.73	464.98									
	1200.00	1196.00	6.81	0.30	85.28	718.76									
	1175.00	1172.00	6.92	0.20	89.76	1137.84									
	1150.00	1148.00	7.04	0.10	73.35	1860.86									
	1125.00	1124.00	7.16	0.05	61.51	3127.40									
	1100.00	1100.00	7.28	0.05	103.95	5379.65									
**Viscometer calibration**	**RPM**	**m**	**b**												
$\eta = m \cdot \tau + b$	100.00	0.03	0.18												
	40.00	0.07	0.44												
	20.00	0.13	0.63												
	10.00	0.26	0.67												
	5.00	0.52	0.59												
	2.50	1.04	-0.63												
	1.20	2.14	-1.23												
	0.60	4.24	-2.37												
	0.30	8.59	-13.63												
	0.20	13.21	-47.98												
	0.10	26.07	-51.37												
	0.05	53.07	-136.87												
$T_{real} = m \cdot T_{set} + b$		0.96	44.50												

1

Supplementary Table 2: Viscometry data including compositional analyses of samples, viscosity measurements, and instrument calibration.

Scale / Parameter	Symbol / Formula	Units	Lower Estimate	Reference Value	Upper Estimate	Comments
**Physical Parameters**						
**Gravity**	g_0	m/s²	-	9.81	-	standard value
**Liquid density**	ρ_0^ℓ	kg/m³	3600	3800	4000	range indicated by alphaMELTS model
**Liquid shear viscosity**	η_0^ℓ	Pas	0.01	0.1	1	extrapolated from Fe-rich, Si-poor silicate melts after *Giordano* et al. [2008]
**Gas density**	ρ_0^g	kg/m³	164.05	205.07	246.08	mixed volatile vapour at 100 MPa, 900 °C.
**Gas compressibility**	β_0	1/Pa	1E-07	1E-06	1E-05	inferred from ideal gas law within reasonable P,T-range
**Water concentration**	$c_0^{H_2O}$	wt%	0.50	1.00	2.00	inferred from experimental petrology and thermodynamic modelling
**Exsolution productivity**	$\gamma_0 = c_0^{H_2O}/(L_0 g_0 \cos\alpha)$	kg/m³/Pa	1.15E-07	2.71E-07	6.73E-07	corresponding to linear exsolution of H_2O in ore liquid on ascent to surface
**Fracture length**	L_0	m	3500	4000	4500	consistent with assumed geometry in volcano deformation models
**Fracture opening**	H_0	m	0.0001	0.001	0.01	assuming flow along thin fractures
**Magma body height**	D_0	m	400	500	600	consistent with assumed geometry in volcano deformation models
**Magma bulk modulus**	K_0	Pa	1E+09	3E+09	1E+10	range indicated by alphaMELTS model
**Rock shear modulus**	G_0	Pa	1E+10	3E+10	1E+11	typical range for silicate rocks
**Fracture angle**	α	deg	10	20	30	inferred from volcano deformation models
**Fault surface**	A_0	m²	6.5E+07	7E+07	7.5E+07	approximate area of truncated elliptical cone of appropriate dimensions
**Slip distance**	d_0	m	0.01	0.03	0.1	assuming collapse events suddenly accommodate longer term deflation
**Bubble fraction**	ϕ_0	vol	0.05	0.1	0.20	reasonable values well below dense packing
**Collapse Event**						
**Moment magnitude**	$M_w = \frac{2}{3}\log_{10}(G_0 A_0 d_0) - 6.06$	-	4.48	5.14	5.86	calculated from fault surface area and slip distance
**Volumetric strain**	$v_0 = d_0/D_0$	1/s	1.67E-05	6.00E-05	2.50E-04	calculated from vertical shortening of magma body by slip distance
**Pressure jump**	$\Delta P_0 = K_0 v_0$	Pa	1.67E+04	1.80E+05	2.50E+06	calculated from volumetric strain and magma bulk modulus
**Fracture Flow**						
**Induced pressure drop**	$F_c = \Delta P_0/(\sin\alpha\ L_0)$	Pa/m	2.13E+01	1.32E+02	1.43E+03	calculated from collapse-related pressure jump projected onto fracture
**Hydrostatic pres. drop**	$F_0 = \rho_0^\ell g_0 \cos\alpha$	Pa/m	3.06E+04	3.50E+04	3.86E+04	calculated from buoyancy of ore-forming liquid projected onto fracture
**Inflow speed**	$w_{in} = F_c H_0^2/(2\eta_0^\ell)$	m/s	1.07E-07	6.58E-04	7.14E+00	calculated from plane-Poisseuille solution for flow along narrow fracture
**Growth number**	$\Lambda = L_0\left(\phi_0(1-\phi_0)F_0(\beta_0 + \gamma_0/\rho_0^g)\right)$	-	**0.5108**	**12.6274**	**278.3498**	dimensionless group governing along-fracture growth of ascent speed

1

Supplementary Table 3: Characteristic scales for appropriate range of parameter values used in our scaling analysis of collapse-driven bubbly fracture flow.
